# Giant thermally induced band-gap renormalization in anharmonic silver chalcohalide antiperovskites†

**DOI:** 10.1039/d5tc00863h

**Published:** 2025-04-14

**Authors:** Pol Benítez, Siyu Chen, Ruoshi Jiang, Cibrán López, Josep-Lluís Tamarit, Jorge Íñiguez-González, Edgardo Saucedo, Bartomeu Monserrat, Claudio Cazorla

**Affiliations:** a Group of Characterization of Materials, Departament de Física, Universitat Politècnica de Catalunya Campus Diagonal Besòs, Av. Eduard Maristany 10–14 08019 Barcelona Spain pol.benitez@upc.edu claudio.cazorla@upc.edu; b Research Center in Multiscale Science and Engineering, Universitat Politècnica de Catalunya Campus Diagonal-Besòs, Av. Eduard Maristany 10–14 08019 Barcelona Spain; c Department of Materials Science and Metallurgy, University of Cambridge Cambridge CB30FS UK; d Cavendish Laboratory, University of Cambridge Cambridge CB30HE UK; e Materials Research and Technology Department, Luxembourg Institute of Science and Technology (LIST) Avenue des Hauts-Fourneaux 5 L-4362 Esch/Alzette Luxembourg; f Department of Physics and Materials Science, University of Luxembourg 41 Rue du Brill L-4422 Belvaux Luxembourg; g Micro and Nanotechnologies Group, Emerging Thin Film Photovoltaics Lab, Departament dEnginyeria Electrònica, Universitat Politècnica de Catalunya Campus Diagonal Besòs, Av. Eduard Maristany 10–14 08019 Barcelona Spain

## Abstract

Silver chalcohalide antiperovskites (CAP), Ag_3_XY (X = S, Se; Y = Br, I), are a family of highly anharmonic inorganic compounds with great potential for energy applications. However, a substantial and unresolved discrepancy exists between the optoelectronic properties predicted by theoretical first-principles methods and those measured experimentally at room temperature, hindering the fundamental understanding and rational engineering of CAP. In this work, we employ density functional theory, tight-binding calculations, and anharmonic Fröhlich theory to investigate the optoelectronic properties of CAP at finite temperatures. Near room temperature, we observe a giant band-gap (*E*_g_) reduction of approximately 20–60% relative to the value calculated at *T* = 0 K, bringing the estimated *E*_g_ into excellent agreement with experimental measurements. This relative *T*-induced band-gap renormalization is roughly twice the largest value previously reported in the literature for similar temperature ranges. Low-energy optical polar phonon modes, which break inversion symmetry and enhance the overlap between silver and chalcogen s electronic orbitals in the conduction band, are identified as the primary drivers of this significant *E*_g_ reduction. Furthermore, when temperature effects are considered, the optical absorption coefficient of CAP increases by nearly an order of magnitude in the visible light spectrum. These findings not only bridge a critical gap between theory and experiment but also pave the way for future technologies where temperature, electric fields, and light dynamically modulate optoelectronic properties, establishing CAP as a versatile platform for energy and photonic applications.

## Abstract 2

Silver chalcohalide antiperovskites (CAP), Ag_3_XY (X = S, Se; Y = Br, I), are a family of highly anharmonic inorganic compounds with great potential for energy applications.

## Graphical abstract

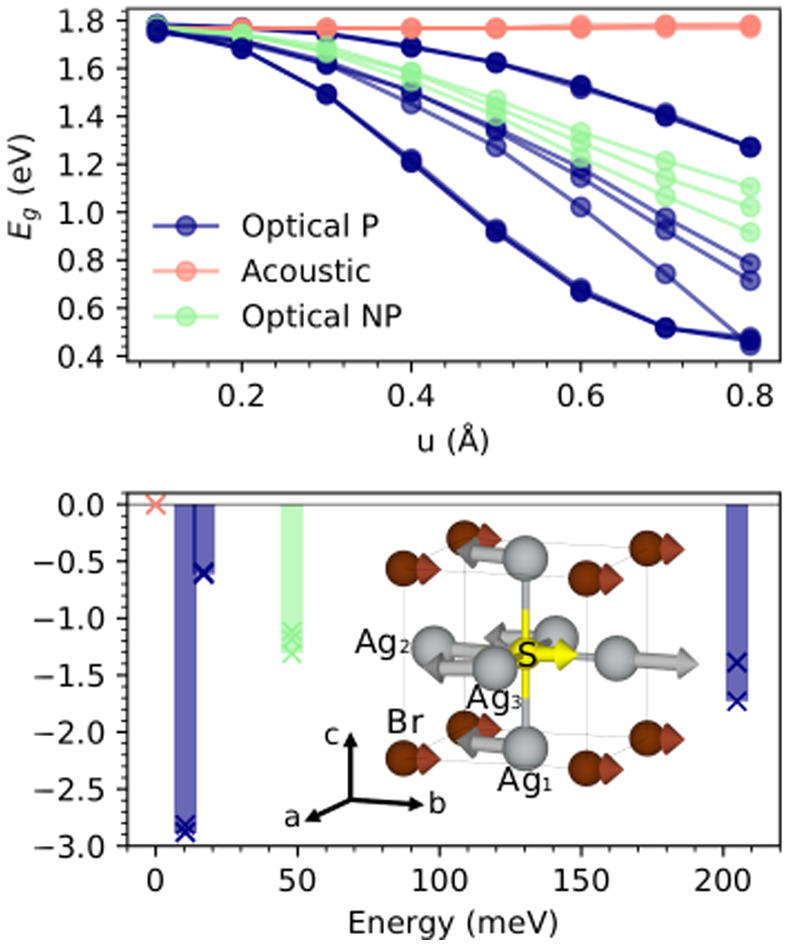

## Introduction

Electron–phonon coupling (EPC), arising from the interactions between electrons and lattice vibrations, is ubiquitous in materials and is responsible for a wide range of condensed matter physical effects.^1–4^ For example, EPC plays a crucial role in the temperature (*T*) dependence of electrical resistivity in metals, carrier mobility in semiconductors, optical absorption in indirect band gap semiconductors, and the onset of conventional superconductivity. Additionally, EPC enables the thermalization of hot carriers, influences the phonon dispersion in metals, and determines the *T*-dependence of electronic energy bands in solids.^5,6^

Likewise, the band gap (*E*_g_) of semiconducting and dielectric materials can be significantly affected by EPC, typically decreasing with increasing temperature (the so-called Varshni effect^7^). This common *E*_g_ behavior can be explained by the Allen–Heine–Cardona perturbative theory, which attributes it to a larger *T*-induced energy increase in the valence band compared to the conduction band due to a greater sensitivity to phonon population variations (*i.e.*, larger second-order electron–phonon coupling constants).^8–10^ Representative examples of this thermal *E*_g_ dependence include diamond, which exhibits a ∼5% band-gap reduction at around 1000 K;^11,12^ antimony sulfide (Sb_2_S_3_), which shows a *E*_g_ reduction of 200 meV in the temperature range of 10 ≤ *T* ≤ 300 K;^13^ MgO, which displays a band-gap reduction of ∼15% in the temperature interval 0 ≤ *T* ≤ 1500 K;^14^ SrTiO_3_, which exhibits a ∼15% band-gap reduction from 300 to 1000 K;^15^ and molecular crystals, which display record band-gap reductions of 15–20% at low temperatures.^16^ Anomalous band-gap thermal behaviour, in which *E*_g_ increases with increasing temperature, has also been observed in a variety of materials such as black phosphorus,^17^ halide perovskites,^18^ and chalcopyrite^19^ and hydride^12^ compounds.

Highly anharmonic silver chalcohalide antiperovskites (CAP)^20^ with chemical formula Ag_3_XY (X = S, Se; Y = Br, I) are structurally similar to lead halide perovskites (*e.g.*, CsPbI_3_), with the “anti” designation indicating the exchange of anions and cations compared to the typical ionic perovskite arrangement. Analogous to lead halide perovskites, CAP are highly promising materials for energy and optoelectronic applications,^21–26^ offering low toxicity due to their lead-free composition.^27,28^ The two most extensively studied CAP compounds, Ag_3_SBr and Ag_3_SI, possess experimentally determined band gaps of approximately 1.0 eV,^29,30^ making them favorable for photovoltaic applications. These materials have also been recognized as high temperature superionic conductors.^21,22^ Additionally, CAP have been investigated as potential thermoelectric materials^25,26^ owing to their substantial vibrational anharmonicity and unique charge transport properties.^23,24^

Intriguingly, for both Ag_3_SBr and Ag_3_SI, there is an enormous disagreement between the *E*_g_ predicted by first-principles methods (at *T* = 0 K, under static lattice conditions) and those measured experimentally at room temperature. In particular, high-level density functional theory (DFT) calculations employing hybrid functionals and including spin–orbit coupling (SOC) effects estimate the band gap of these two archetypal CAP to be 1.8 and 1.4 eV, respectively.^29–31^ The *E*_g_ discrepancies between theory and measurements amount to 60–80% of the experimental values (*i.e.*, differences of 0.5–0.8 eV), which are unusually large and call for a careful inspection of the factors causing them.

In this study, we assessed the influence of EPC effects on the *E*_g_ and optical absorption spectra of CAP using first-principles DFT methods, tight-binding calculations, and anharmonic Fröhlich theory. Near room temperature, our computational investigations revealed a giant *E*_g_ reduction of 20–60% relative to the value calculated at *T* = 0 K, bringing the estimated band gap into excellent agreement with the experimental values. Low-energy optical polar phonons, which cause large symmetry-breaking structural distortions and promote the overlap between silver and chalcogen s electronic orbitals in the conduction band, were identified to be the primary mechanism driving this substantial *T*-induced band-gap reduction. Furthermore, at finite temperatures the optical absorption spectra of CAP were significantly enhanced, in some cases by nearly an order of magnitude. The polar nature of the phonons causing these effects opens up new technological possibilities, where the optoelectronic properties of materials could be effectively manipulated by external electric fields and light.

## Results

**Fig. 1:**
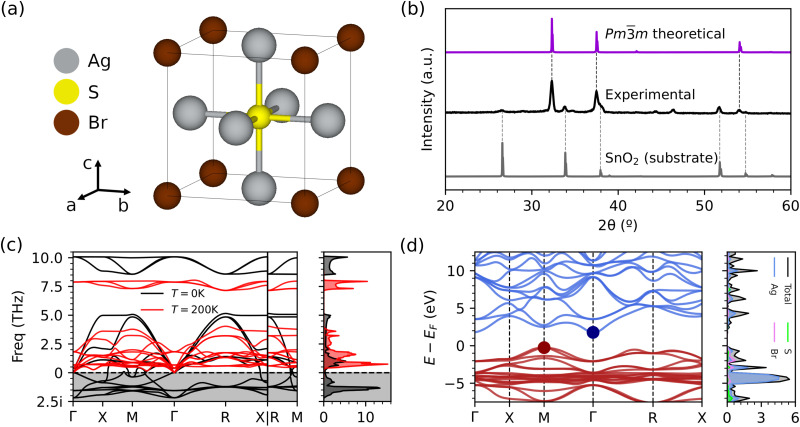
General physical properties of the archetypal CAP Ag_3_SBr. (a) The cubic *Pm*3̄*m* phase experimentally observed at room temperature. (b) Experimental diffractogram of Ag_3_SBr^30^ compared with the theoretical one estimated for the cubic *Pm*3̄*m* phase. (c) Vibrational phonon spectrum (left) and phonon density of states (right) calculated within the harmonic approximation for the cubic *Pm*3̄*m* phase of Ag_3_SBr at *T* = 0 K (black lines) and at *T* = 200 K (red lines) fully considering anharmonic effects. (d) Electronic band structure (left) and density of states (right) of Ag_3_SBr calculated with the hybrid functional HSEsol.^35^ Red and blue lines (dots) represent valence (top of the valence) and conduction (bottom of the conduction) bands, respectively.

The room-temperature phase of both Ag_3_SI and Ag_3_SBr have been experimentally identified as cubic with the space group *Pm*3̄*m*.^30,32–34^ This phase is characterized by a five-atom unit cell: a chalcogen atom at the center of the cube, halide atoms in each vertex, and silver atoms at the center of each face (Fig. 1a and b). Phonon calculations of this phase within the harmonic approximation (*T* = 0 K) reveal imaginary phonon branches, thus indicating dynamical instability. However, when phonons are calculated fully accounting for anharmonic effects at finite-*T* conditions, the resulting phonon spectrum is well-behaved with no signs of instability (Fig. 1c).^20^ Thus, the cubic *Pm*3̄*m* phase was considered throughout this work for all CAP.

As discussed in the Introduction, the discrepancies between the experimentally measured (at *T* = 300 K) and theoretically determined (at *T* = 0 K) band gaps of Ag_3_SBr and Ag_3_SI are tremendously large (*i.e.*, 60–80% of the experimental values). Therefore, we investigated their potential causes by assessing the impact of electron–phonon coupling (EPC) and temperature on the band gap of CAP.

Band gap renormalization due to electron–phonon interactions is typically estimated using two main approaches: density functional perturbation theory (DFPT)^1^ and finite-differences.^2^ In DFPT, electron–phonon interactions are treated as a perturbation, with band energy variations (and consequently, band gap shifts) derived from the Fan-Migdal and Debye–Waller self-energies, as described by Allen and Heine.^36^ A key advantage of DFPT is its computational efficiency, as it does not require the use of supercells. This method has been widely employed in band gap renormalization studies^37^ and is implemented in popular *ab initio* codes such as EPW.^38^

Finite-differences approaches, on the other hand, are computationally more demanding, requiring supercells to reproduce phonons in real space and dynamical simulations to correctly sample the electronic response to ionic fluctuations. However, they offer distinct advantages over DFPT. One key benefit is their flexibility, as they can be applied with any underlying electronic structure method. Additionally, finite-differences methods naturally incorporate terms beyond the lowest order in the electron–phonon interaction, making them particularly useful for capturing higher-order effects.^18^ Readers seeking a more comprehensive discussion of DFPT and finite-differences methods are referred to the review articles,^1,2^ which extensively cover these techniques.

In this study, we employ the finite-differences approach to estimate temperature-renormalized band gaps, as CAP materials exhibit strong anharmonicity.^20^ Consequently, renormalizing their electronic band energies at the harmonic level would be inadequate. Moreover, since accurate CAP band gap predictions require the use of hybrid functionals and spin–orbit coupling, the finite-differences approach emerges as the most practical and reliable choice.

In particular, we performed first-principles calculations and *ab initio* molecular dynamics (AIMD) simulations based on DFT (Methods). Additionally, to capture long-range EPC effects, we employed anharmonic Fröhlich theory^39–42^ considering long-range dipole–dipole interactions and *T*-renormalized phonons (Methods). Furthermore, the optical absorption spectra of all CAP were assessed at *T* ≠ 0 K conditions and the main EPC mechanisms underlying the *E*_g_ discrepancies were identified with the help of a tight-binding model.

### EPC band-gap renormalization in CAP

The *T*-renormalized band gap of CAP was calculated as follows:

1 *E*_g_(*T*) = *E*_g_(0) + Δ*E*_g_(*T*),

where *E*_g_(0) represents the static band gap and the correction term Δ*E*_g_ can be expressed as the sum of short-(S) and long-wavelength (L) phonon contributions:^40^

2 Δ*E*_g_(*T*) = Δ*E*^S^_g_(*T*) + Δ*E*^L^_g_(*T*).

The short-wavelength phonon correction was estimated through AIMD simulations using a supercell (Methods), where the band-gap value was averaged over multiple generated configurations, as described in ref. 39:3
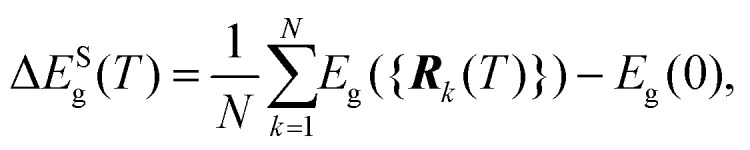
where *N* represents the total number of considered configurations and {***R***_*k*_} the atomic position of the *k*-th configuration. To achieve accurate band-gap corrections, it was essential to use hybrid functionals and incorporate spin–orbit coupling effects (Supplementary methods, ESI†), which significantly increase the computational effort. Extensive numerical tests were conducted to optimize the supercell size, *k*-point mesh, and value of *N*, ensuring that these critical effects were accurately captured in the calculations (Methods and Supplementary methods, ESI†).

In polar materials, there is an additional contribution to the band-gap renormalization stemming from long-range Fröhlich coupling that is not fully captured by the finite size of the supercells employed in the AIMD simulations.^39–43^ This long-wavelength phonon band-gap correction can be expressed as follows:4
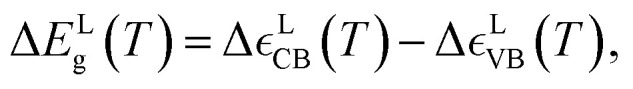
where CB and VB refer to the bottom conduction and top valence band levels, respectively.

For a 3D polar material, the *T*-induced energy level shifts appearing in eqn (4) can be computed as follows:^39^5
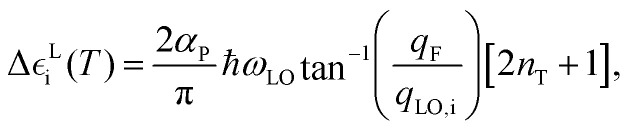
where *α*_P_ represents the polaron constant, *ω*_LO_ the phonon frequency averaged over the three longitudinal optical Γ phonon modes,^43^ and *q*_F_ a truncation factor. The truncation factor *q*_F_ can be approximated as the Debye sphere radius and *q*_LO,i_ is defined as 
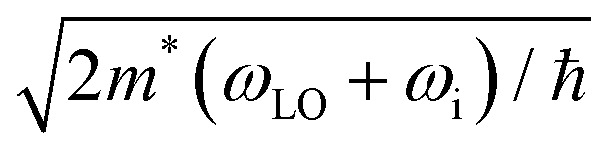
, m* being the charge carrier effective mass and *ħω*_*i*_ the state energy. The term *n*_T_ is the Bose–Einstein occupation number corresponding to the average LO vibrational frequency, and the polaron constant can be computed as follows:^39^6
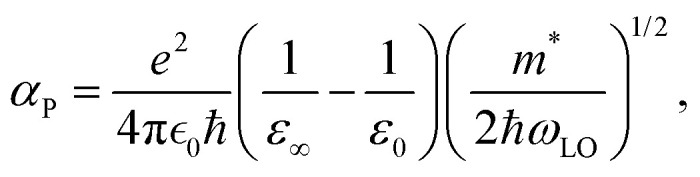
where *ε*_∞_ is the high-frequency dielectric constant and *ε*_0_ the static permittivity of the system. Quantum nuclear effects have been disregarded throughout this work, hence the *T*-induced energy level shifts in eqn (5) were offset by their zero-temperature values Δ*ε*^L^_*i*_(0).

**Fig. 2:**
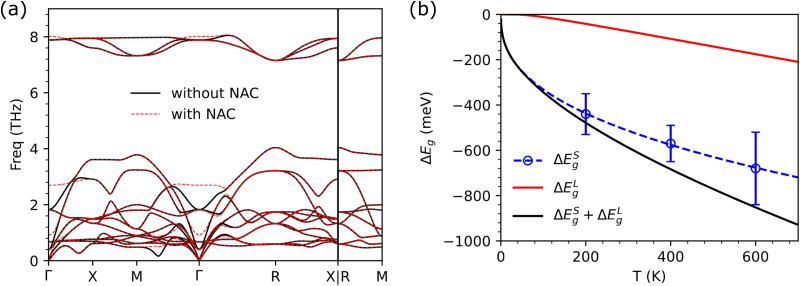
Anharmonic phonon spectrum and thermal band-gap corrections estimated for the archetypal CAP Ag_3_SBr. (a) Anharmonic phonon spectrum obtained at *T* = 200 K neglecting (black solid lines) and considering (red dashed lines) non-analytical corrections (NAC). (b) Short- and long-wavelength phonon band-gap corrections, Δ*E*^S^_g_ and Δ*E*^L^_g_, respectively, expressed as a function of temperature (excluding quantum nuclear effects). The short-range correction term was evaluated at several temperature points (blue circles and error bars); as a guide to the eye, the Δ*E*^S^_g_ data points were fitted to an arbitrary polynomial function (blue dashed line). Calculations were performed at the HSEsol + SOC level.^35^

Fig. 2a presents the anharmonic phonon spectrum calculated for Ag_3_SBr under finite-temperature conditions, accounting for long-range dipole–dipole interactions (*i.e.*, including non-analytical corrections), which result in LO–TO splitting near the reciprocal space point Γ. Fig. 2b shows the corresponding short- and long-wavelength phonon band-gap corrections expressed as a function of temperature, which are always negative. Since in this study the Δ*E*^L^_g_ correction term has been calculated using the material's anharmonic phonon spectrum, we refer to this method as anharmonic Fröhlich theory (Methods).

In Fig. 2b, it is observed that near room temperature the Δ*E*^S^_g_ correction is dominant and significantly larger than Δ*E*^L^_g_, approximately six times greater in the absolute value. Notably, at *T* = 400 K, the total band-gap correction for Ag_3_SBr amounts to 0.7 eV, which is of giant proportions, representing roughly 40% of the *E*_g_ value calculated at zero temperature (excluding quantum nuclear effects).

**Fig. 3:**
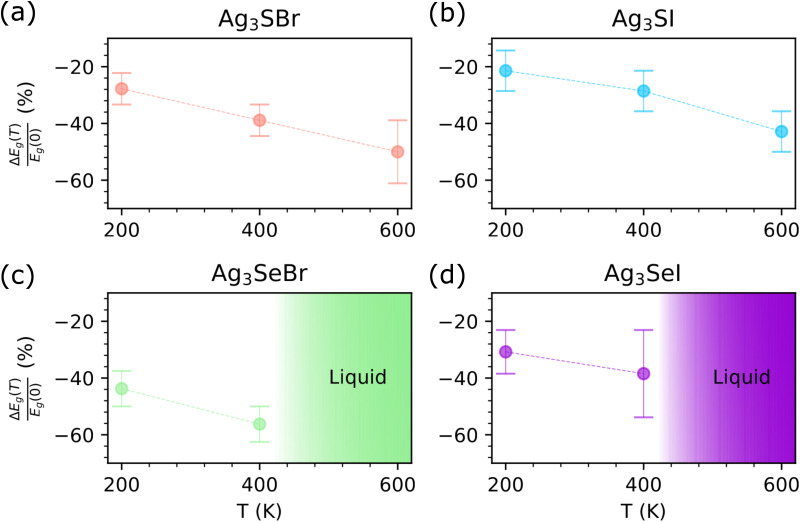
Temperature-induced relative band-gap variation in CAP. Percentages are referenced to the band gap calculated at *T* = 0 K conditions (excluding quantum nuclear effects), namely, Δ*E*_g_(*T*) = *E*_g_(*T*) − *E*_g_(0), for (a) Ag_3_SBr, (b) Ag_3_SI, (c) Ag_3_SeBr, and (d) Ag_3_SeI. Error bars indicate numerical uncertainties and dashed lines are a guide to the eye. Shaded areas indicate regions of thermodynamic stability of the liquid phase (theory). Calculations were performed at the HSEsol + SOC level.^35^

Fig. 3 shows the relative band-gap variation, referenced to the value calculated at zero temperature and expressed as a function of temperature, for the four CAP compounds Ag_3_SBr, Ag_3_SI, Ag_3_SeBr and Ag_3_SeI. In all cases, the band gap significantly decreases as the temperature increases (Table 1). The relative *T*-induced *E*_g_ reduction is largest for Ag_3_SeBr and smallest for Ag_3_SI. In particular, near room temperature, the band gap of Ag_3_SBr and Ag_3_SI is reduced by 39% and 29% while those of Ag_3_SeBr and Ag_3_SeI decrease by 56% and 38%, respectively (Fig. 3). As shown in Table 1, the agreement between the experimental and theoretical *E*_g_ values for Ag_3_SBr and Ag_3_SI improves as the temperature increases. In Ag_3_SeBr and Ag_3_SeI, the liquid phase is stabilized over the crystal phase at moderate temperatures (Fig. 3c and d); thus no band gaps were estimated for these two compounds under *T* > 400 K conditions.

**Table 1:** Theoretical band gaps of CAP as a function of temperature. *E*_g_ values were obtained at zero temperature (excluding quantum nuclear effects) at *T* = 200, 400 and 600 K. Calculations were performed at the HSEsol + SOC level.^35^ Numerical uncertainties are provided, which mainly result from the Δ*E*^S^_g_ correction term. Short- and long-wavelength phonon band-gap corrections, Δ*E*^S^_g_ and Δ*E*^L^_g_, respectively, are provided at each temperature (excluding quantum nuclear effects). The experimental band gaps measured at room temperature for Ag_3_SBr and Ag_3_SI^30^ are shown for comparison

CAP	*E* ^0K^_g_ [eV]	*E* ^200K^_g_ [eV]	Δ*E*^S^_g_ [meV]	Δ*E*^L^_g_ [meV]	*E* ^400K^_g_ [eV]	Δ*E*^S^_g_ [meV]	Δ*E*^L^_g_ [meV]	*E* ^600K^_g_ [eV]	Δ*E*^S^_g_ [meV]	Δ*E*^L^_g_ [meV]	*E* ^exp^_g_ [eV]
Ag_3_SBr	1.8	1.3 ± 0.1	−440	−42	1.1 ± 0.1	−570	−108	0.9 ± 0.2	−680	−175	1.0
Ag_3_SI	1.4	1.1 ± 0.1	−260	−29	1.0 ± 0.1	−290	−75	0.8 ± 0.1	−490	−122	0.9
Ag_3_SeBr	1.6	0.9 ± 0.1	−630	−42	0.7 ± 0.1	−770	−105	Liquid	—	—	—
Ag_3_SeI	1.3	0.9 ± 0.1	−370	−37	0.8 ± 0.2	−400	−90	Liquid	—	—	—

Notably, our theoretical *E*_g_ results obtained at *T* = 400 K are fully consistent with the available experimental data obtained at room temperature. This excellent agreement near ambient conditions strongly suggests that the neglect of EPC effects is the main reason for the huge theoretical–experimental *E*_g_ discrepancies discussed in the Introduction. The *T*-induced relative band-gap renormalization found in CAP are of giant proportions, ranging from 20 to 60% near room temperature, setting a new record previously held by molecular crystals, which exhibited a 15 to 20% band-gap renormalization for similar temperature ranges.^16^

Table 1 also presents the value of the Δ*E*^S^_g_ and Δ*E*^L^_g_ correction terms estimated for each CAP at three different temperatures. In all cases, both the short- and long-wavelength phonon corrections are negative, with the former term considerably surpassing the latter in absolute value. For example, at *T* = 400 K, the short-range band-gap corrections are seven and four times larger than the long-range ones calculated for Ag_3_SeBr and Ag_3_SI, respectively. As the temperature is raised, the size of the two band-gap correction terms increases in absolute value, with |Δ*E*^L^_g_| exhibiting the largest relative enhancement (*e.g.*, approximately a 320% relative increase for Ag_3_SBr from 200 to 600 K).

To provide further insights into the impact of thermal effects on the electronic band structure of CAP, we also examined how band morphology evolves with temperature. Since band-gap renormalization in this study is assessed using the finite-differences approach based on AIMD simulations, it is convenient to unfold the energy bands calculated for the supercell into the reciprocal space of the primitive unit cell.^44^ This was done using the Easyunfold software,^45^ focusing on the representative CAP compound Ag_3_SBr (Fig. S1, ESI†).

Specifically, we analyzed five uncorrelated supercell snapshots extracted from a long AIMD trajectory (∼100 ps) at *T* = 200 K. For this particular case, the PBEsol exchange–correlation functional was employed due to the very high computational cost of performing hybrid functional calculations on supercells. Reassuringly, we verified that the band structures obtained using semilocal and hybrid functionals are practically equivalent in morphology (Fig. S2, ESI†).

Our results indicate that thermal effects lead to a downward shift of the conduction band minimum, which remains located at the Γ point, which is consistent with the band-gap trends observed in the corresponding electronic density of states. However, the ionic disorder induced by lattice vibrations causes a noticeable flattening of the valence band near its top. As a result, the valence band maximum becomes poorly defined, unlike in the static case (Fig. S1, ESI†). The consistency of results across the five ionically disordered configurations suggests that this small sampling is sufficient to capture the key temperature-induced changes in band morphology.

### Thermal effects on the optical absorption coefficient of CAP

Following a similar approach to that used for the calculation of *T*-renormalized band gaps (*i.e.*, performing AIMD simulations with a supercell and averaging the quantity of interest over several of the generated configurations), we determined the frequency-dependent complex dielectric tensor of CAP under *T* ≠ 0 K conditions (Methods and Supplementary methods, ESI†), employing linear response theory. From the average dielectric tensor, we computed several macroscopic optical properties like the optical absorption coefficient, *α*(*ω*), refractive index, and reflectivity.^46^

**Fig. 4:**
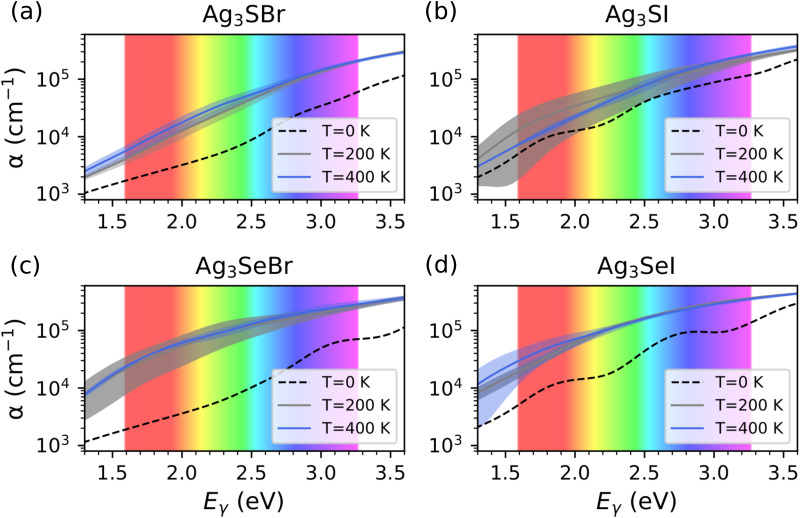
Optical absorption coefficient (*α*) of CAP calculated at different temperatures as a function of photon energy. (a) Ag_3_SBr, (b) Ag_3_SI, (c) Ag_3_SeBr, and (d) Ag_3_SeI. Solid lines represent the estimated average values and statistical errors are indicated with shaded thick curves. The rainbow-colored region denotes photons with energy in the visible spectrum. Calculations were performed at the HSEsol + SOC level.^35^

Fig. 4 shows the optical absorption spectra estimated for CAP as a function of incident light wavelength and temperature. It is found that *α* is significantly enhanced under increasing temperature, in some cases by as much as an order of magnitude. Similarly to the band gap, the *T*-induced optical absorption variations are the largest for Ag_3_SeBr (for which *α* ∼ 10^3^–10^5^ cm^−1^ at zero temperature and ∼10^4^–10^6^ cm^−1^ at 200 K) and smallest for Ag_3_SI (for which *α* ∼ 10^3^–10^5^ cm^−1^ at any temperature). It is also noted that the most significant optical absorption changes generally occur at low temperatures, that is, within the 0 ≤ *T* ≤ 200 K interval. These *T*-induced *α* trends align well with the remarkably large influence of the EPC on the band gap, underscoring the critical role of thermal renormalization effects on the optoelectronic properties of CAP.

Unfortunately, we cannot directly compare our theoretical *α*(*ω*) results with experimental data, as such data are not available in the literature. Notably, Caño *et al.*^30^ measured the optical absorption coefficient of CAP films scaled by their layer thickness, *d*, specifically, *ᾱ* ≡ *α*·*d*. However, since the thickness of the synthesized CAP films was not determined in work,^30^ we cannot access the physical quantity of interest. In this regard, performing new optoelectronic experiments on CAP films across a broad range of temperatures, including the low-*T* regime, would be highly desirable.

### EPC mechanisms in CAP

We have already shown that temperature and EPC effects are essential for understanding the thermal evolution of the optoelectronic properties of CAP and for achieving a consistent agreement between first-principles calculations and room-temperature experiments. Next, we focus on unraveling the primary ionic–electronic mechanisms underlying these key temperature and EPC effects.

**Fig. 5:**
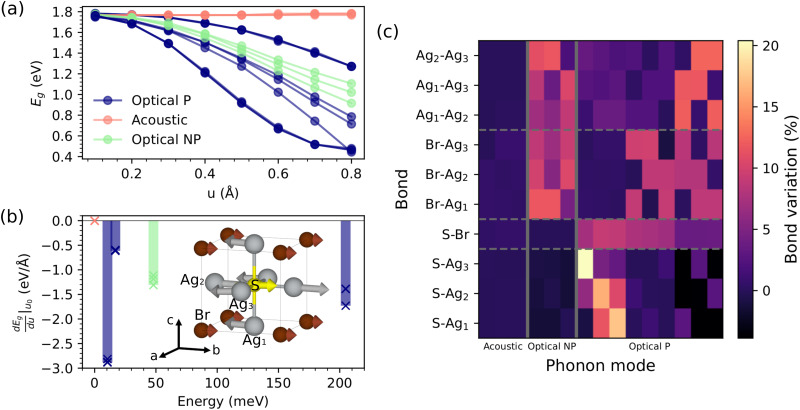
Phonon-induced band-gap variation estimated for the archetypal CAP Ag_3_SBr. (a) Band gap as a function of the lattice distortion amplitude *u* for acoustic, polar optical (P) and non-polar optical (NP) Γ phonons. (b) Derivative of the band gap with respect to the phonon distortion amplitude calculated at *u*_0_ = 0.4 Å and expressed as a function of the phonon energy. The eigenmode of the optical polar Γ phonon rendering the largest band-gap derivative in absolute value is sketched: Ag, S and Br atoms are represented with grey, yellow and brown spheres, respectively. Calculations were performed at the HSEsol + SOC level.^35^ (c) Γ phonon-induced relative bond length distortions in the cubic *Pm*3̄*m* phase for a distortion amplitude of 0.4 Å.

As shown in Fig. 5a, the influence of each of the fifteen Γ phonon modes on the band gap of Ag_3_SBr was analysed by monitoring the change in *E*_g_ driven by frozen-phonon eigenmode distortions of increasing amplitude, *u*. The Γ phonons were classified into acoustic (A), optical polar (P) and optical nonpolar (NP), where the P phonons break the inversion symmetry of the centrosymmetric cubic *Pm*3̄*m* phase. It was found that *E*_g_ is unresponsive to acoustic phonon distortions, as expected, while optical P phonons produce the largest band-gap variations. As the amplitude of the optical phonon distortions increases, *E*_g_ systematically decreases in both the P and NP cases.

Fig. 5b shows the value of the derivative of the band gap with respect to the phonon distortion amplitude, *u*, expressed as a function of the phonon eigenmode energy (as obtained from *T*-renormalised phonon calculations, Methods). We found that low-energy polar phonon modes (∼10 meV) cause the most significant band-gap reductions, followed by high-energy lattice vibrations of the same type (∼200 meV). At room temperature, phonon excitations with the lowest energy host the highest populations and, consequently, represent the most characteristic lattice vibrations in the crystal. Therefore, based on the results shown in Fig. 3 and 5, we conclude that low-energy polar phonon modes are primarily responsible for the substantial temperature-induced *E*_g_ reduction reported in this study for CAP compounds.

The eigenmode of the optical P phonon with the lowest energy is represented in Fig. 5b. As observed therein, this frozen-phonon lattice distortion reduces the distance between the central sulfur atom and one adjacent silver atom (Ag2), while increasing the other two S–Ag1 and S–Ag3 bond lengths, compared to the undistorted cubic unit cell. Fig. 5c summarizes the relative bond length variation, in absolute value, for all pairs of atoms resulting from each of the fifteen Γ phonon modes calculated for the cubic *Pm*3̄*m* phase. As shown therein, the optical P phonons produce the largest S–Ag distance changes (up to 20%), while the optical NP phonons cause the largest Br–Ag bond length variations (up to 12%). The Br–Ag bond lengths are also appreciably impacted by the optical P phonons (5–10%). This general behaviour is reminiscent of that observed for optical polar phonons in model perovskite oxides like BaTiO_3_ (with atomic substitutions Ag ↔ O, S ↔ Ti and Br ↔ Ba).^47,48^

**Fig. 6:**
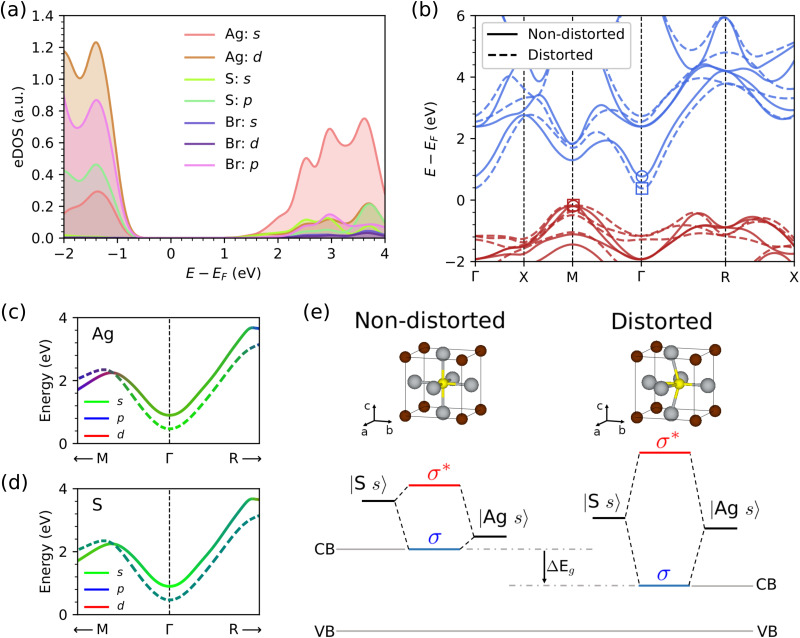
Electronic band structure properties of the archetypal CAP Ag_3_SBr. (a) Electronic density of states calculated for the equilibrium cubic *Pm*3̄*m* phase. Calculations were performed at the HSEsol + SOC level.^35^ (b) Electronic band structure calculated for the equilibrium and phonon distorted (*i.e.*, considering the lowest-energy Γ optical P mode with amplitude *u*_0_ = 0.4 Å) cubic *Pm*3̄*m* phase; in both cases, the energy bands are referred to a same energy origin, a deep core electronic level that remains unaffected by the distortion (Methods). (c) and (d) The conduction band near its minimum at Γ computed with a TB model for silver and sulfur atoms (Methods); solid and dashed lines correspond to the equilibrium and distorted structures, respectively. (e) Sketch illustrating the mechanism of band-gap closure induced by low-energy polar soft phonon modes in CAP. Upon phonon distortion, the hybridization of silver and sulfur s electrons in the conduction band is enhanced, lowering (increasing) the energy of the corresponding bonding σ (antibonding σ*) state.

After identifying the phonon modes that underpin the giant *T*-induced band-gap reduction reported in this study for CAP, specifically low-energy optical P modes, we further analyse the induced changes in the electronic band structure. Fig. 6a shows the electronic density of states calculated for the archetypal compound Ag_3_SBr (equilibrium geometry). It is observed that the top of the valence band (VB) is dominated by highly hybridized silver d and chalcohalide p electronic orbitals, while the bottom of the conduction band (CB) is dominated by isotropic and more delocalized S and Ag s orbitals. The electronic band structure in Fig. 6b shows that the VB corresponds to the high-symmetry reciprocal space point M (1/2,1/2,0), while the CB to the center of the Brillouin zone, Γ (0,0,0); thus the band gap of Ag_3_SBr is indirect (we have checked that the same conclusion applies to the rest of CAP compounds analyzed in this study).

The effects on the electronic band structure resulting from a frozen-phonon lattice distortion corresponding to the lowest-energy optical P eigenmode (*u*_0_ = 0.4 Å) are twofold (Fig. 6b). First, due to the breaking of phonon-induced inversion symmetry, the energy band degeneracy at the reciprocal space point M is lifted. However, the band gap of the system is unaffected by this energy degeneracy lifting effect since the VB remains practically invariant. Second, the CB edge experiences a significant decrease in energy and, as a consequence, the band gap of the system is reduced by approximately 30%. Therefore, we may conclude that the giant *T*-induced *E*_g_ reduction reported in this study for CAP is primarily caused by low-energy polar phonon modes that induce a pronounced CB energy decrease.

To better understand the electronic origins of the optical P phonon-induced CB energy lowering, we constructed a tight-binding (TB) model based on Wannier functions that accurately reproduces our DFT band structure results (Methods and Fig. S3, ESI†). Specifically, the TB model consists of s, p, and d orbitals for the five atoms in the unit cell, resulting in a total of 45 distinct Wannier orbitals. Consistently, the TB model reproduces the dominant Ag and S s character of the CB and its energy lowering under the polar lattice distortion of interest (Fig. 6c and d).

According to this TB model, the impact of the frozen-phonon distortion on the Ag and S s conduction orbitals is twofold. First, the difference in their kinetic energies, corresponding to the diagonal Hamiltonian matrix elements difference |〈Ag s|*H*|Ag s〉 − 〈S s|*H*|S s〉|, decreases (Fig. S3, ESI†). And second, the hopping s term involving the Ag2 and S atoms, represented by the off-diagonal Hamiltonian matrix element 〈Ag2 s|*H*|S s〉, increases (Fig. S3, ESI†). The general physical interpretation that follows from these TB results is that the polar frozen-phonon distortion enhances the hybridization of Ag2 and S s conduction orbitals, which lowers and increases the energy of the corresponding bonding (σ) and antibonding (σ*) states, respectively. Consequently, the CB, which is dominated by the Ag2-S σ interaction, is lowered. The revealed EPC mechanism, which overall produces a band-gap reduction, is schematically represented in Fig. 6e.

## Discussion

Thermal expansion is another physical mechanism that can influence the band gap of materials,^40^ but it was not explicitly considered in this study due to its high computational cost. However, we performed a series of tests in which we arbitrarily increased and decreased the unit cell volume of Ag_3_SBr and estimated the corresponding relative *E*_g_ variation (Fig. S4 and Table S1, ESI†). We found that, when the volume of the system increases by a reasonable ∼1%, as might occur due to thermal expansion near room temperature, the band gap decreases by only ∼40 meV (*i.e.*, an order of magnitude smaller change than short-wavelength phonon corrections, Table 1). Additionally, thermal expansion likely contributes to the softening of optical P modes, increasing their population and consequently enhancing the *E*_g_ reduction due to EPC. Therefore, while thermal expansion effects were omitted in our calculations, the reported results likely represent a lower bound of the actual effect, and our conclusions for CAP compounds remain robust and accurate.

One may wonder whether, in addition to silver chalcohalide antiperovskites, there exist other families of materials exhibiting similarly large *T*-renormalization effects on the band gap and optical absorption coefficient. As discussed in previous sections, the polar nature of low-energy optical phonons appears to be essential in this regard. Consequently, a tentative set of necessary conditions for identifying potential materials that display similar *T*-induced effects on the optoelectronic properties may include dielectric materials exhibiting (1) centrosymmetric crystalline phases, (2) low-energy or even imaginary optical polar phonons, and (3) highly hybridized and delocalized electronic orbitals near the Fermi energy level. The availability of large DFT calculations and phonon databases may enable high-throughput material screening of such a kind.^49,50^

Ferroelectric oxide perovskites, exemplified by the archetypal compounds SrTiO_3_ (STO) and BaTiO_3_ (BTO), appear to satisfy the set of necessary conditions outlined above. Notably, a significant band-gap modulation has been reported for STO under biaxial strain conditions, although this phenomenon arises from different physical mechanisms than those identified in this study for CAP compounds (*i.e.*, energy degeneracy lifting due to symmetry breaking).^51^ Moreover, the experimental room-temperature band gap of BTO (≈3.2 eV^52^) shows substantial disagreement with zero-temperature theoretical estimates obtained with hybrid functionals (≈4.0 eV^53^), highlighting an experiment–theory inconsistency similar to that described for Ag_3_SBr and Ag_3_SI in the Introduction. Additionally, the band gap of the multiferroic oxide perovskite BiFeO_3_ exhibits a remarkable temperature-dependent shrinkage, decreasing by approximately 50% within the temperature range 300 ≤ *T* ≤ 1200 K,^54^ likely influenced by the magnetic degrees of freedom.^55^ These findings suggest that the temperature effects and EPC mechanisms identified in this study for CAP compounds may have broader relevance, potentially extending to other well-known families of functional materials. Theoretical investigations exploring this possibility are currently underway.

The polar nature of the optical phonon modes, which cause the significant *T*-induced reduction in the *E*_g_ of CAP, opens up exciting technological possibilities. Similar to how an electric field can stabilize a polar phase with ferroelectric polarization over a paraelectric state at constant temperature through a phase transformation,^56^ it is likely that polar optical phonons in CAP can also be stimulated using external electric fields. This possibility implies that the optoelectronic properties of CAP could be effectively tuned by applying an electric field rather than altering the temperature, providing a more practical approach for the development of advanced optical devices and other technological applications. Experimental validation of this hypothesis would be highly valuable.

Finally, advances in light sources and time-resolved spectroscopy have made it possible to excite specific atomic vibrations in solids and to observe the resulting changes in their electronic and electron–phonon coupling properties.^57–59^ These developments also suggest the possibility of tuning the optoelectronic properties of CAP, as well as of similar materials like oxide perovskites,^60–62^ through specific phonon excitations using optical means such as lasers. This approach may simplify the design and manufacture of practical setups by eliminating the need for electrode deposition. Therefore, the results presented in this work are significant not only from a fundamental perspective but also for envisioning potential technological applications in which the optical and electronic properties of materials could be effectively tuned by external fields and photoexcitation.

## Conclusions

In this study, we have explored the temperature effects on the band gap of silver chalcohalide antiperovskites (CAP), specifically Ag_3_XY compounds (X = S, Se; Y = Br, I), which are promising for energy and photonic applications due to their lead-free composition, high ionic conductivity, and optoelectronic properties. The key findings of our research are summarized as follows. A giant reduction in the band gap of CAP materials has been disclosed at room temperature, ranging from 20% to 60% relative to their values calculated at zero temperature (neglecting zero-point corrections). This large band-gap renormalization brings theoretical predictions closer to experimental results, resolving previous discrepancies.

The significant *E*_g_ reduction is attributed to strong electron–phonon coupling driven by low-energy polar phonon modes, which distorts the lattice symmetry, increases the overlap between silver and chalcogen *s* orbitals in the conduction band, and lowers the reference energy of the resulting bonding state. With increasing temperature, the optical absorption coefficient of CAP materials also rises, enhancing their response to visible light by nearly an order of magnitude and highlighting their potential for optoelectronic applications.

This research demonstrates that CAP compounds exhibit giant band-gap renormalization primarily due to temperature and electron–phonon coupling effects, suggesting that they could be tailored for specific applications through thermal, electric field, and/or optical control. These fundamental findings open possibilities for the design of innovative optoelectronic devices and establish a foundation for exploring similar effects in other dielectric materials with strong electron–phonon coupling.

## Methods

### Zero-temperature first-principles calculations

The DFT calculations^63–65^ were performed using the semilocal PBEsol approximation,^66^ considering the following electronic states as valences: Ag 5s–4d, S 3s–3p, Se 4s–4p, Br 4s–4p, I 5s–5p. Wave functions were represented in a plane-wave basis set truncated at 650 eV. Using these parameters and a dense *k*-point grid of 8 × 8 × 8 for reciprocal-space Brillouin zone (BZ) sampling, we obtained zero-temperature energies converged to within 0.5 meV per formula unit. For the geometry relaxations, a force tolerance of 0.005 eV Å^−1^ was imposed on all the atoms. The optoelectronic properties were estimated using hybrid functionals^35^ (Fig. S5 and Supplementary methods, ESI†) and considering spin–orbit coupling (SOC) effects, a computational approach named HSEsol + SOC here. In this case, to make the calculations feasible, the energy cutoff and *k*-point grid were slightly reduced to 550 eV and 6 × 6 × 6, respectively. Quantum nuclear effects^65^ were disregarded throughout this work, as well as the likely presence of weak excitons.^67,68^

All-electron DFT calculations were also performed with the WIEN2k package^69^ using the local-density approximation (LDA)^70^ to the exchange correlation energy along using the linearized augmented plane wave method (FP-LAPW).^71,72^ The technical parameters for these calculations were a 10 × 10 × 10 *k*-point grid and a muffin-tin radius equal to *R*_MT_ = 7.0/*K*_max_, where *K*_max_ represents the plane-wave cutoff. Localized energy-resolved Wannier states^73^ were then obtained for the tight-binding calculations^74–76^ considering the relevant Hilbert space in the interval −10 ≤ *E* ≤ 20 eV around the Fermi energy.

### Finite-temperature first-principles simulations

*Ab initio* molecular dynamics (AIMD) simulations were performed in the canonical (*N*, *V*, *T*) ensemble, neglecting thermal expansion effects and employing a large simulation cell containing 320 atoms with periodic boundary conditions applied along the three Cartesian directions. The temperature in the AIMD simulations was kept fluctuating around a set-point value by using Nose–Hoover thermostats. Newton's equations of motion were integrated using the standard Verlet's algorithm with a time step of 1.5 × 10^−3^ ps. Γ-Point sampling for reciprocal-space integration was employed in the AIMD simulations, which spanned approximately 100 ps. These calculations were performed with the semilocal PBEsol exchange–correlation functional.^66^

### Harmonic phonon calculations

The second-order interatomic force constant matrix of all CAP and resulting harmonic phonon spectrum were calculated using the finite-differences method as is implemented in the PhonoPy software.^77^ Large supercells (*i.e.*, 4 × 4 × 4 for the cubic *Pm*3̄*m* supercell containing 320 atoms) and a dense *k*-point grid of 3 × 3 × 3 for BZ sampling were employed for the phonon calculations of targeted structures. Several numerical tests were conducted that demonstrated the adequacy of the selected *k*-point grid. These calculations were performed with the semilocal PBEsol exchange–correlation functional.^66^

### Anharmonic phonon calculations

The DynaPhoPy code^78^ was used to calculate the anharmonic lattice dynamics (*i.e.*, *T*-renormalized phonons) of CAP in the cubic *Pm*3̄*m* phase from *ab initio* molecular dynamics (AIMD) simulations. A reduced 2 × 2 × 2 supercell and 4 × 4 × 4 *k*-point grid for BZ sampling were employed in the AIMD simulations to maintain high numerical accuracy (Supplementary methods and work, ESI,† (ref. 20)).

A normal-mode decomposition technique was employed in which the atomic velocities **v**_*jl*_(*t*) (*j* and *l* represent particle and Cartesian direction indexes) generated during fixed-temperature AIMD simulation runs were expressed as follows:7
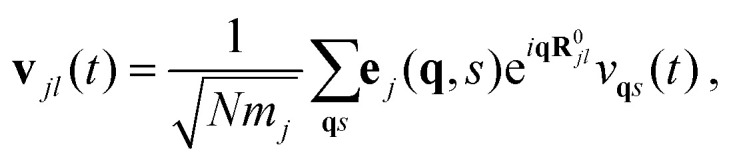
where *N* is the number of particles, *m*_*j*_ the mass of particle *j*, **e**_*j*_(**q**,*s*) a phonon mode eigenvector (**q** and *s* stand for the wave vector and phonon branch), **R**^0^_*jl*_ the equilibrium position of particle *j*, and *v*_**q***s*_ the velocity of the corresponding phonon quasiparticle.

The Fourier transform of the autocorrelation function of *v*_**q***s*_ was then calculated, yielding the following power spectrum:8
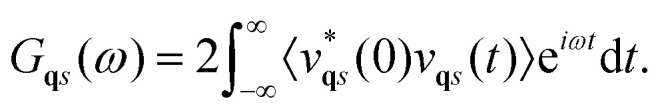


Finally, this power spectrum was approximated by a Lorentzian function of the following form:9
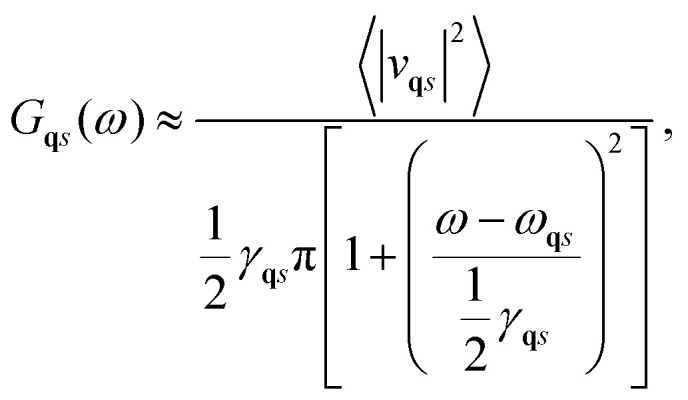
from which a *T*-renormalized quasiparticle phonon frequency, *ω*_**q***s*_(*T*), was determined as the peak position, and the corresponding phonon linewidth, *γ*_**q***s*_(*T*), as the full width at half maximum. These calculations were performed with the semilocal PBEsol exchange–correlation functional.^66^

### Short-wavelength phonon band-gap correction

The electron–phonon correction to the band gap due to the short-range phonon modes was computed as the difference between the band gap at zero temperature for the static structure and the average band gap obtained from AIMD simulations performed with a supercell, namely10
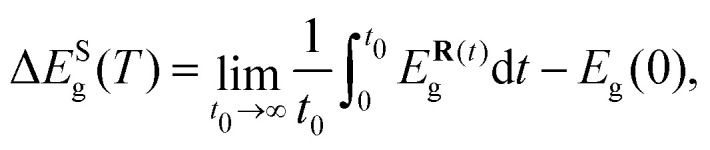
where **R** represents the positions of the atoms in the supercell at a given time *t* of the AIMD simulation. This expression can be numerically approximated as shown in eqn (3), where the band gap is averaged over a finite number, *N*, of configurations. Similarly, thermal effects on the dielectric tensor were computed.

These calculations were performed using the hybrid HSEsol exchange–correlation functional^35^ and considering spin–orbit coupling effects (HSEsol + SOC). Due to the high computational expense involved, the AIMD calculations were performed using a supercell containing 40 atoms and a *k*-point mesh of a single point. The total number of configurations used for the average was *N* = 10 for each material and temperature. These values were found to be appropriate for obtaining band-gap results accurate to within 0.1 eV (Supplementary methods, ESI†).

### Long-wavelength phonon band-gap correction

The electron–phonon correction to the band gap due to long-range phonon modes was computed using the Fröhlich equation for a three-dimensional polar material.^39–42^ This correction was determined as the difference in the shifts of the conduction and valence bands, as given in eqn (4), where the shift of each band was computed using the expression in eqn (5). The physical quantities entering this latter expression were determined with DFT methods; the electron and hole effective masses were computed using the parabolic approximation at the maximum of the valence band and the minimum of the conduction band, respectively. For the LO phonon frequency, we used an effective value computed as the average of the three corresponding modes, as done in ref. 43. Temperature-induced anharmonic effects were fully taken into account for the calculation of these LO phonon frequencies, using the renormalized phonon frequencies obtained at *T* = 200 K. Due the superionic behavior of these materials at higher temperatures, the renormalized phonon dispersions calculated at *T* ≥ 200 K conditions presented imaginary frequencies,^20^ and hence were not considered in this study.

The high-frequency and static dielectric constants calculated for the cubic *Pm*3̄*m* phase were unusually large in static calculations, since this phase is not vibrationally stable at *T* = 0 K. Thus, the dielectric constants were recomputed as functions of temperature (using the same approach as we applied to the band gap and dielectric tensor) to capture thermal effects. In view of their weak temperature dependence, and for simplicity, the dielectric constants obtained at *T* = 200 K were employed throughout this work. We also considered the anisotropy of the dielectric tensor using the following formula:11
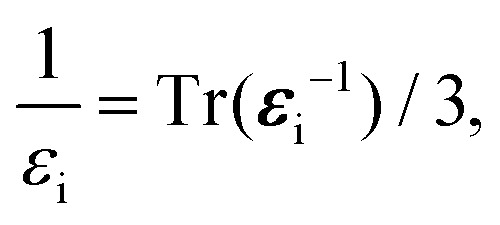
where *ε* represents the dielectric constant associated with the dielectric tensor ***ε***.

## Data availability

The data supporting this article have been included as part of the ESI.†

## Conflicts of interest

There are no conflicts to declare.

## Supplementary Material

TC-013-D5TC00863H-s001

## References

[cit1] Giustino F. (2017). Electron-phonon interactions from first principles. Rev. Mod. Phys..

[cit2] Monserrat B. (2018). Electron-phonon coupling from finite differences. J. Phys.: Condens. Matter.

[cit3] Lin Z., Zhigilei L. V., Celli V. (2008). Electron-phonon coupling and electron heat capacity of metals under conditions of strong electron-phonon nonequilibrium. Phys. Rev. B:Condens. Matter Mater. Phys..

[cit4] Bohnen K.-P., Heid R., Renker B. (2001). Phonon dispersion and electron-phonon coupling in MgB_2_ and AlB_2_. Phys. Rev. Lett..

[cit5] Monserrat B., Park J.-S., Walsh A. (2018). Role of electron-phonon coupling and thermal expansion on band gaps, carrier mobility, and interfacial offsets in kesterite thin-film solar cells. Appl. Phys. Lett..

[cit6] Monserrat B., Vanderbilt D. (2017). Temperature dependence of the bulk Rashba splitting in the bismuth tellurohalides. Phys. Rev. Mater..

[cit7] Varshni Y. P. (1967). Temperature dependence of the energy gap in semiconductors. Physica.

[cit8] O'Donnell K. P., Chen X. (1991). Temperature dependence of semiconductor band gaps. Appl. Phys. Lett..

[cit9] Poncé S., Gillet Y., Janssen J. L., Marini A., Verstraete M., Gonze X. (2015). Temperature dependence of the electronic structure of semiconductors and insulators. J. Chem. Phys..

[cit10] Park J., Saidi W. A., Chorpening B., Duan Y. (2022). Applicability of Allen-Heine-Cardona theory on MO_*x*_ metal oxides and ABO_3_ perovskites: Toward high-temperature optoelectronic applications. Chem. Mater..

[cit11] Giustino F., Louie S. G., Cohen M. L. (2010). Electron-phonon renormalization of the direct band gap of diamond. Phys. Rev. Lett..

[cit12] Monserrat B., Drummond N. D., Needs R. J. (2013). Anharmonic vibrational properties in periodic systems: energy, electron-phonon coupling, and stress. Phys. Rev. B:Condens. Matter Mater. Phys..

[cit13] Liu Y., Monserrat B., Wiktor J. (2023). Strong electron-phonon coupling and bipolarons in Sb_2_*S*_3_. Phys. Rev. Mater..

[cit14] Zhang Y., Wang Z., Xi J., Yang J. (2020). Temperature-dependent band gaps in several semiconductors: from the role of electron-phonon renormalization. J. Phys.: Condens. Matter.

[cit15] Wu Y.-N., Saidi W. A., Wuenschell J. K., Tadano T., Ohodnicki P., Chorpening B., Duan Y. (2020). Anharmonicity explains temperature renormalization effects of the band gap in SrTiO_3_. J. Phys. Chem. Lett..

[cit16] Monserrat B., Engel E. A., Needs R. J. (2015). Giant electron-phonon interactions in molecular crystals and the importance of nonquadratic coupling. Phys. Rev. B:Condens. Matter Mater. Phys..

[cit17] Villegas C. E. P., Rocha A. R., Marini A. (2016). Anomalous temperature dependence of the band gap in black phosphorus. Nano Lett..

[cit18] Saidi W. A., Poncé S., Monserrat B. (2016). Temperature dependence of the energy levels of methylammonium lead iodide perovskite from first-principles. J. Phys. Chem. Lett..

[cit19] Artus L., Bertrand Y. (1987). Anomalous temperature dependence of fundamental gap of AgGaS_2_ and AgGaSe_2_ chalcopyrite compounds. Solid State Commun..

[cit20] Benítez P., López C., Liu C., Caño I., Tamarit J.-L., Saucedo E., Cazorla C. (2025). Crystal structure prediction and phase stability in highly anharmonic silver-based chalcohalide anti-perovskites. PRX Energy.

[cit21] Takahashi T., Yamamoto O. (1966). The Ag/Ag_3_*SI*/*I*_2_ solid-electrolyte cell. Electrochim. Acta.

[cit22] Hull S. (2004). Superionics: crystal structures and conduction processes. Rep. Prog. Phys..

[cit23] Wakamura K., Miura F., Kojima A., Kanashiro T. (1990). Observation of anomalously increasing phonon damping constant in the β phase of the fast-ionic conductor Ag_3_SI. Phys. Rev. B:Condens. Matter Mater. Phys..

[cit24] Sakuma T. (1985). Treatment of anharmonic thermal vibration by using transformation of scattering vector. J. Phys. Soc. Jpn..

[cit25] Kawamura J., Shimoji M., Hoshino H. (1981). The ionic conductivity and thermoelectric power of the superionic conductor Ag_3_SBr. J. Phys. Soc. Jpn..

[cit26] Magistris A., Pezzati E., Sinistri C. (1972). Thermoelectric properties of high-conductivity solid electrolytes. Z. Naturforsch..

[cit27] Palazon F. (2022). Metal chalcohalides: Next generation photovoltaic materials?. Sol. RRL.

[cit28] Ghorpade U. V., Suryawanshi M. P., Green M. A., Wu T., Hao X., Ryan K. M. (2023). Emerging chalcohalide materials for energy applications. Chem. Rev..

[cit29] Sebastiá-Luna L., Rodkey N., Mirza A. S., Mertens S., Lal S., Melchor A., Carranza G., Calbo J., Righetto M., Sessolo M., Herz L. M., Vandewal K., Ortí E., Morales-Masis M., Bolink H. J., Palazon F. (2023). Chalcohalide antiperovskite thin films with visible light absorption and high charge-carrier mobility processed by solvent-free and low-temperature methods. Chem. Mater..

[cit30] Caño I., Turnley J. W., Benítez P., López-Álvarez C., Asensi J.-M., Payno D., Puigdollers J., Placidi M., Cazorla C., Agrawal R., Saucedo E. (2024). Novel synthesis of semiconductor chalcohalide anti-perovskites by low-temperature molecular precursor ink deposition methodologies. J. Mater. Chem. C.

[cit31] Liu Z., Mi R., Ji G., Liu Y., Fu P., Hu S., Xia B., Xiao Z. (2021). Bandgap engineering and thermodynamic stability of oxyhalide and chalcohalide antiperovskites. Ceram. Int..

[cit32] Sakuma T., Hoshino S. (1980). The phase transition and the structures of superionic conductor Ag_3_SBr. J. Phys. Soc. Jpn..

[cit33] Hoshino S., Fujishita H., Takashige M., Sakuma T. (1981). Phase transition of Ag_3_ SX (X= I, Br). Solid State Ionics.

[cit34] Cho N., Kikkawa S., Kanamaru F., Yoshiasa A. (1994). Structural refinement of Ag_3_SI by single crystal X-ray diffraction method. Solid State Ionics.

[cit35] Krukau A. V., Vydrov O. A., Izmaylov A. F., Scuseria G. E. (2006). Influence of the exchange screening parameter on the performance of screened hybrid functionals. J. Chem. Phys..

[cit36] Allen P. B., Heine V. (1976). Theory of the temperature dependence of electronic band structures. J. Phys. C: Solid State Phys..

[cit37] Cannuccia E., Marini A. (2011). Effect of the quantum zero-point atomic motion on the optical and electronic properties of ciamond and trans-polyacetylene. Phys. Rev. Lett..

[cit38] Lee H., Poncé S., Bushick K. (2023). *et al.*, Electron-phonon physics from first principles using the EPW code. npj Comput. Mater..

[cit39] Zacharias M., Scheffler M., Carbogno C. (2020). Fully anharmonic nonperturbative theory of vibronically renormalized electronic band structures. Phys. Rev. B.

[cit40] Chen S., Parker I. J., Monserrat B. (2024). Temperature effects in topological insulators of transition metal dichalcogenide monolayers. Phys. Rev. B.

[cit41] Poncé S., Gillet Y., Janssen J. L., Marini A., Verstraete M., Gonze X. (2015). Temperature dependence of the electronic structure of semiconductors and insulators. J. Chem. Phys..

[cit42] Zacharias M., Giustino F. (2020). Theory of the special displacement method for electronic structure calculations at finite temperature. Phys. Rev. Res..

[cit43] Melo P. M., Abreu J. C., Guster B., Giantomassi M., Zanolli Z., Gonze X., Verstraete M. J. (2023). High-throughput analysis of Fröhlich-type polaron models. npj Comput. Mater..

[cit44] Popescu V., Zunger A. (2012). Extracting *E* versus *k* effective band structure from supercell calculations on alloys and impurities. Phys. Rev. Res..

[cit45] Zhu B., Kavanagh S. R., Scanlon D. (2024). easyunfold: A Python package for unfolding electronic band structures. J. Open Source Softw..

[cit46] Wang V., Xu N., Liu J. C., Tang G., Geng W. T. (2021). VASPKIT: A user-friendly interface facilitating high-throughput computing and analysis using VASP code. Comput. Phys. Commun..

[cit47] Cohen R. E. (1992). Origin of ferroelectricity in perovskite oxides. Nature.

[cit48] Zhong W., Vanderbilt D., Rabe K. M. (1994). Phase transitions in BaTiO_3_ from first principles. Phys. Rev. B:Condens. Matter Mater. Phys..

[cit49] Jain A., Ong S. P., Hautier G. (2013). *et al.*, The Materials Project: A materials genome approach to accelerating materials innovation. APL Mater..

[cit50] https://github.com/atztogo/phonondb

[cit51] Berger R. F., Fennier C. J., Neaton J. B. (2011). Band gap and edge engineering via ferroic distortion and anisotropic strain: The case of SrTiO_3_. Phys. Rev. Lett..

[cit52] Wemple S. H. (1970). Polarization fluctuations and the optical-absorption edge in BaTiO_3_. Phys. Rev. B.

[cit53] Evarestov R. A., Bandura A. V. (2012). First-principles calculations on the four phases of BaTiO_3_. J. Comput. Chem..

[cit54] Weber M. C., Guennou M., Toulouse C., Cazayous M., Gillet Y., Gonze X., Kreisel J. (2016). Temperature evolution of the band gap in BiFeO_3_ traced by resonant Raman scattering. Phys. Rev. B.

[cit55] Cazorla C., Íñiguez-González J. (2013). Insights into the phase diagram of bismuth ferrite from quasiharmonic free-energy calculations. Phys. Rev. B:Condens. Matter Mater. Phys..

[cit56] Cazorla C., Íñiguez-González J. (2018). Giant direct and inverse electrocaloric effects in multiferroic thin films. Phys. Rev. B.

[cit57] Kennes D., Wilner E., Reichman D. (2017). *et al.*, Transient superconductivity from electronic squeezing of optically pumped phonons. Nat. Phys..

[cit58] Dekorsky T., Kütt W., Pfeifer T., Kurz H. (1993). Coherent control of LO-phonon dynamics in opaque semiconductors by femtosecond laser pulses. Europhys. Lett..

[cit59] Pomarico E., Mitrano M., Bromberger H. (2017). *et al.*, Enhanced electron-phonon coupling in graphene with periodically distorted lattice. Phys. Rev. B.

[cit60] Qi Y., Liu S., Lindenberg A. M., Rappe A. M. (2018). Ultrafast electric field pulse control of giant temperature change in ferroelectrics. Phys. Rev. Lett..

[cit61] Peng B., Hu Y., Murakami S., Zhang T., Monserrat B. (2020). Topological phonons in oxide perovskites controlled by light. Sci. Adv..

[cit62] Rurali R., Escorihuela-Sayalero C., Tamari J.-L., Íñiguez-González J., Cazorla C. (2024). Giant photocaloric effects across a vast temperature range in ferroelectric perovskites. Phys. Rev. Lett..

[cit63] Kresse G., Furthmüller J. (1996). Efficient iterative schemes for ab initio total-energy calculations using a plane-wave basis set. Phys. Rev. B:Condens. Matter Mater. Phys..

[cit64] Blöchl P. E. (1994). Projector augmented-wave method. Phys. Rev. B:Condens. Matter Mater. Phys..

[cit65] Cazorla C., Boronat J. (2017). Simulation and understanding of atomic and molecular quantum crystals. Rev. Mod. Phys..

[cit66] Perdew J. P., Ruzsinszky A., Csonka G. I. (2008). *et al.*, Restoring the density-gradient expansion for exchange in solids and surfaces. Phys. Rev. Lett..

[cit67] Yu P. Y. and Cardona M., Fundamentals of Semiconductors: Physics and Materials Properties, Springer, 2010

[cit68] Liu C., Errea I., Ding C. (2023). *et al.*, Excitonic insulator to superconductor phase transition in ultra-compressed helium. Nat. Commun..

[cit69] Blaha P., Karlheinz S., Fabien T., Laskowski R., Georg M., Laurence D. M. (2020). WIEN2k: An APW + lo program for calculating the properties of solids. J. Chem. Phys..

[cit70] Perdew J. P., Wang Y. (1992). Accurate and simple analytic representation of the electron-gas correlation energy. Phys. Rev. B.

[cit71] David J. S. , Planewaves, pseudopotentials and the LAPW method, Springer, New York, NY, 2006

[cit72] Blaha P., Schwarz K., Sorantin P., Trickey S. B. (1990). Full-potential, linearized augmented plane wave programs for crystalline systems. Comput. Phys. Commun..

[cit73] Marzari N., Vanderbilt D. (1997). Maximally localized generalized Wannier functions for composite energy bands. Phys. Rev. B:Condens. Matter Mater. Phys..

[cit74] Wei K., Rosner H., Pickett W. E., Scalettar R. T. (2002). Insulating ferromagnetism in La_4_Ba_2_Cu_2_O_10_: An ab initio Wannier function analysis. Phys. Rev. Lett..

[cit75] Wei-Guo Y., Volja D., Wei K. (2006). Orbital ordering in LaMnO_3_: Electron-electron versus electron-lattice interactions. Phys. Rev. Lett..

[cit76] Ruoshi J., Lang Z.-J., Berlijn T., Ku W. (2023). Variation of carrier density in semimetals via short-range correlation: A case study with nickelate NdNiO_2_. Phys. Rev. B.

[cit77] Togo A., Tanaka I. (2015). First principles phonon calculations in materials science. Scr. Mater..

[cit78] Carreras A., Togo A., Tanaka I. (2017). DynaPhoPy: A code for extracting phonon quasiparticles from molecular dynamics simulations. Comput. Phys. Commun..

