# Supplementary material for: Giant thermally induced band-gap renormalization in anharmonic silver chalcohalide antiperovskites
Source: J Mater Chem C Mater. 2025 Apr 14;13(20):10399–412. doi: 10.1039/d5tc00863h (PMC12035675; doi:10.1039/d5tc00863h)
Supplement: TC-013-D5TC00863H-s001 [file TC-013-D5TC00863H-s001.pdf]

Supplementary Information (SI) for Journal of Materials Chemistry C.
This journal is © The Royal Society of Chemistry 2025

Supplementary Information for "Giant Thermally Induced Band-Gap Renormalization in Anharmonic Silver Chalcohalide Antiperovskites"

Pol Benítez, Cibrán López, Josep-Lluís Tamarit, and Claudio Cazorla

Group of Characterization of Materials, Departament de Física, Universitat Politècnica de Catalunya, Campus Diagonal Besòs, Av. Eduard Maristany 10–14, 08019 Barcelona, Spain and Research Center in Multiscale Science and Engineering, Universitat Politècnica de Catalunya, Campus Diagonal-Besòs, Av. Eduard Maristany 10–14, 08019 Barcelona, Spain

Siyu Chen and Bartomeu Monserrat

Department of Materials Science and Metallurgy, University of Cambridge, Cambridge CB30FS, UK and Cavendish Laboratory, University of Cambridge, Cambridge CB30HE, UK

Ruoshi Jiang

Department of Materials Science and Metallurgy, University of Cambridge, Cambridge CB30FS, UK

Jorge Íñiguez-González

Materials Research and Technology Department, Luxembourg Institute of Science and Technology (LIST), Avenue des Hauts-Fourneaux 5, L-4362 Esch/Alzette, Luxembourg and Department of Physics and Materials Science, University of Luxembourg, 41 Rue du Brill, L-4422 Belvaux, Luxembourg

Edgardo Saucedo

Research Center in Multiscale Science and Engineering, Universitat Politècnica de Catalunya, Campus Diagonal-Besòs, Av. Eduard Maristany 10–14, 08019 Barcelona, Spain and

Micro and Nanotechnologies Group,
Emerging Thin Film Photovoltaics Lab,
Departament d'Enginyeria Electrònica,
Universitat Politècnica de Catalunya, Campus Diagonal Besòs,
Av. Eduard Maristany 10-14, 08019 Barcelona, Spain.

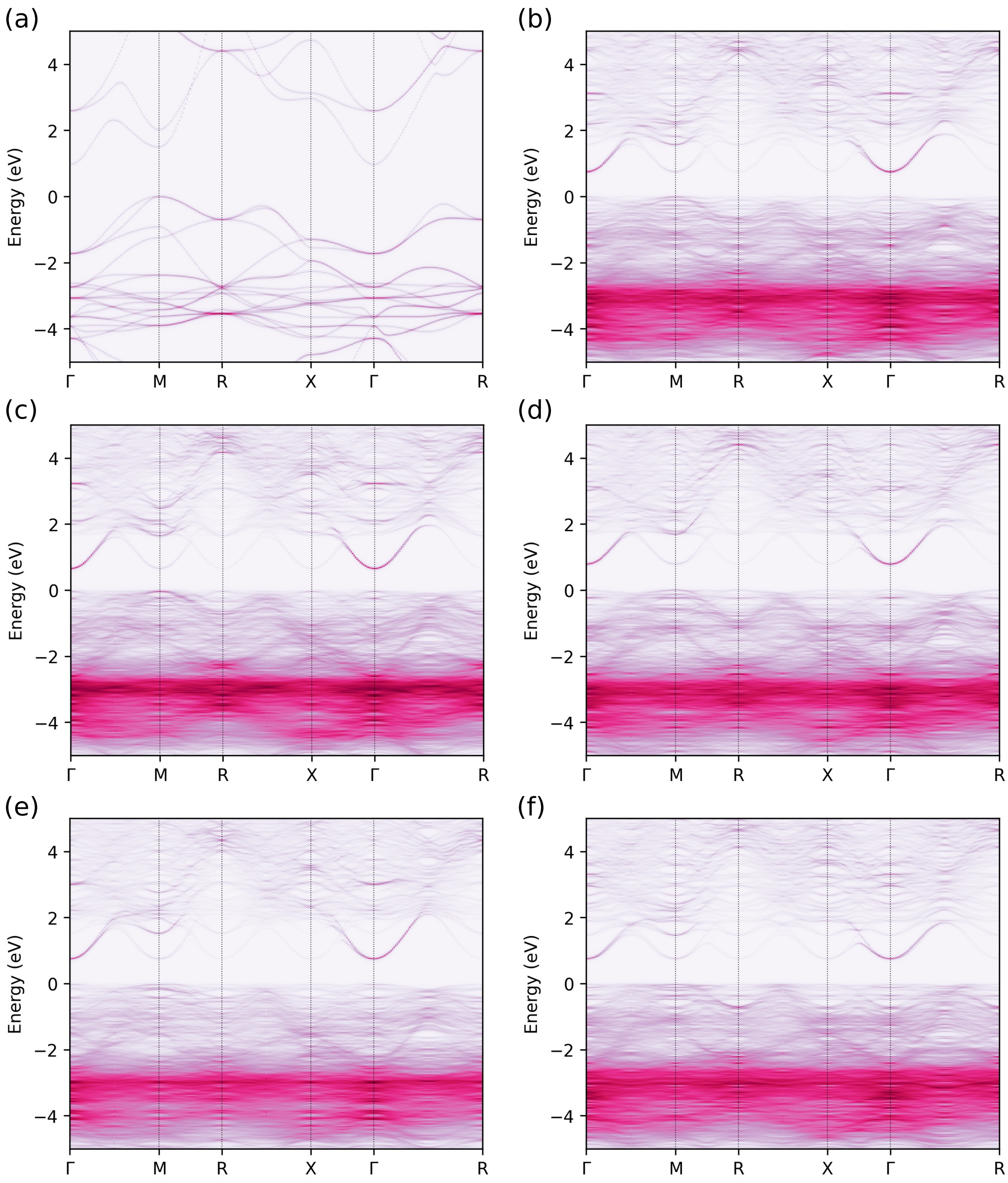

Supplementary Figure 1: (a) Electronic band structure of the perfectly static cubic supercell ($2 \times 2 \times 2$) of Ag_3SBr. (b)–(f) Electronic band structures computed for five uncorrelated Ag_3SBr supercell configurations, unfolded onto the reciprocal space of the unit cell. The five unfolded band structures are qualitatively and quantitatively very similar to each other but display significant differences compared to the static unit cell case.

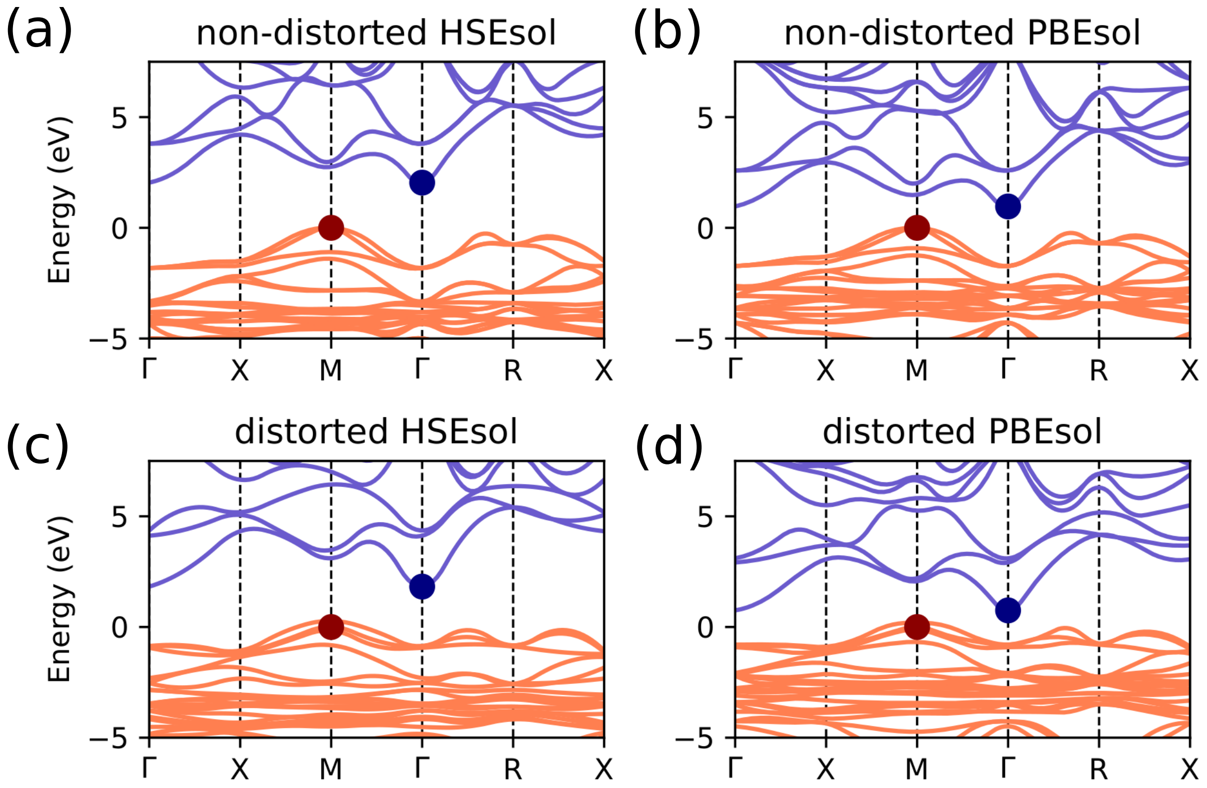

Supplementary Figure 2: Energy bands computed for the cubic $Pm\overline{3}m$ phase of the compound Ag_3SBr, using (a) HSEsol and (b) PBEsol, and for the distorted structure ($u = 0.4$ Å) using (c) HSEsol and (d) PBEsol. Orange lines represent the valence bands, while blue lines represent the conduction bands. The red dot represents the maximum of the valence band, and the blue dot represents the minimum of the conduction band.

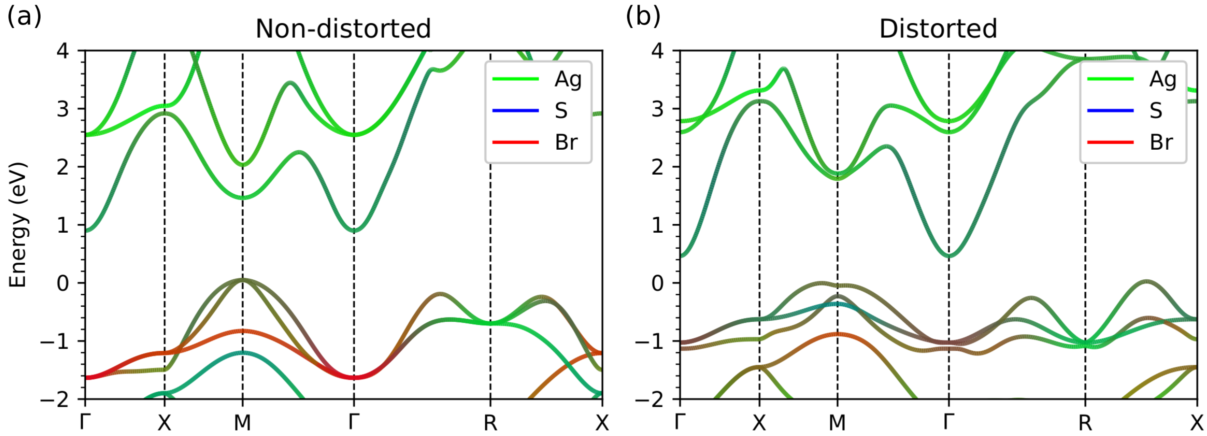

(c) Non-distorted

	$\|Ag_1\ s\rangle$	$\|Ag_2\ s\rangle$	$\|Ag_3\ s\rangle$	$\|S\ s\rangle$
$\langle Ag_1\ s\|$	3.91	0.11	0.11	2.26
$\langle Ag_2\ s\|$	0.11	3.91	0.11	2.26
$\langle Ag_3\ s\|$	0.11	0.11	3.91	2.26
$\langle S\ s\|$	2.26	2.26	2.26	15.24

(d) Distorted

	$\|Ag_1\ s\rangle$	$\|Ag_2\ s\rangle$	$\|Ag_3\ s\rangle$	$\|S\ s\rangle$
$\langle Ag_1\ s\|$	4.10	0.46	0.16	2.25
$\langle Ag_2\ s\|$	0.46	4.35	0.47	2.39
$\langle Ag_3\ s\|$	0.16	0.47	4.10	2.25
$\langle S\ s\|$	2.25	2.39	2.25	14.05

Supplementary Figure 3: Tight-binding (TB) band structure calculated for Ag_3SBr: (a) equilibrium and (b) phonon-distorted structures. The partial contribution of each atomic species is represented by green, blue and red colors for silver, sulfur and bromine ions, respectively. TB Hamiltonian matrix (in eV) calculated for the most relevant orbitals involved in the band-gap closure mechanism revealed in this study for CAP: (c) equilbrium and (d) phonon-distorted structures.

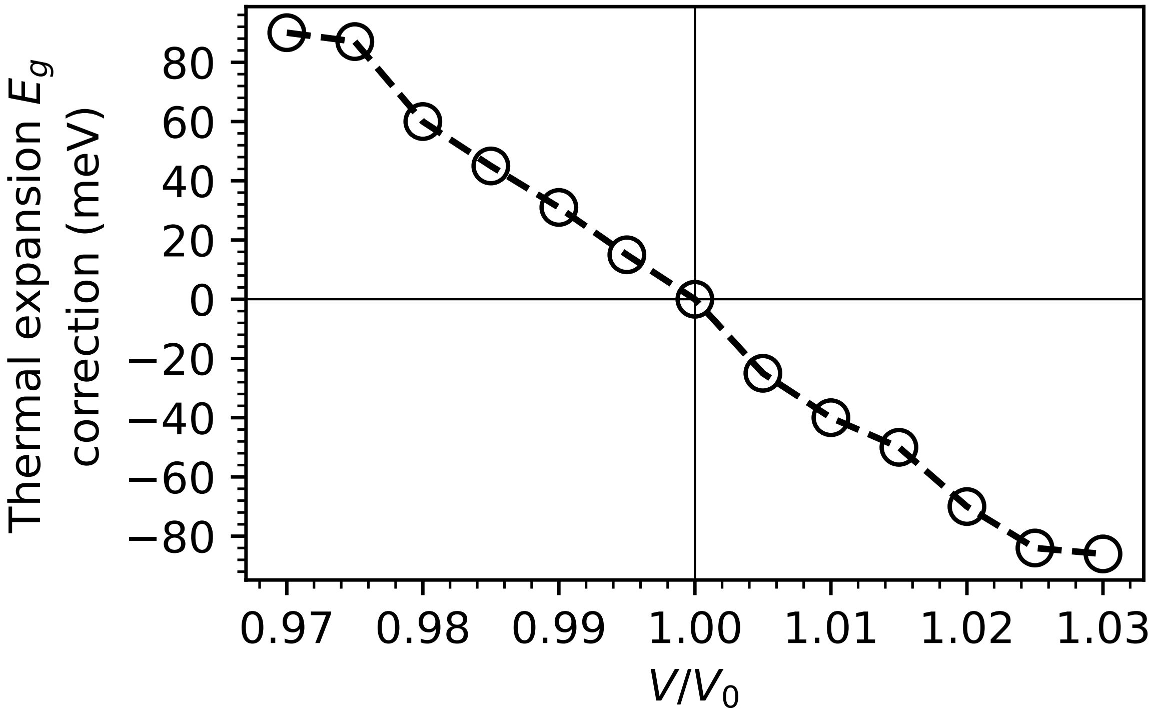

Supplementary Figure 4: Volume expansion and contraction effects on the first-principles estimation of the band gap of Ag_3SBr. When increasing (decreasing) the volume of the system the band gap decreases (increases). Calculations were performed at the HSEsol+SOC level.

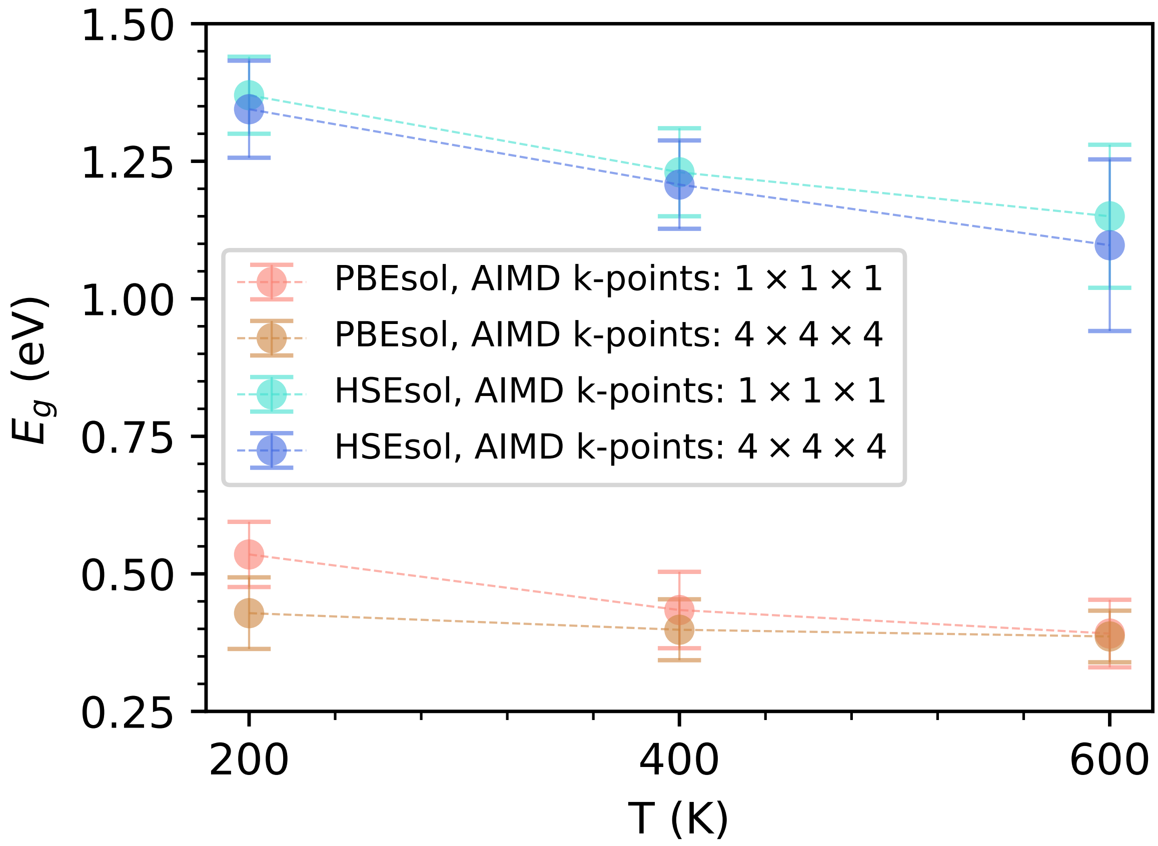

Supplementary Figure 5: Thermally renormalized band gap computed with PBEsol and HSEsol exchange-correlation functionals for the $Pm\overline{3}m$ phase of Ag_3SBr. The averages are taken over states reached with AIMD simulations using a $2 \times 2 \times 2$ supercell with $1 \times 1 \times 1$ and $4 \times 4 \times 4$ **k**-points.

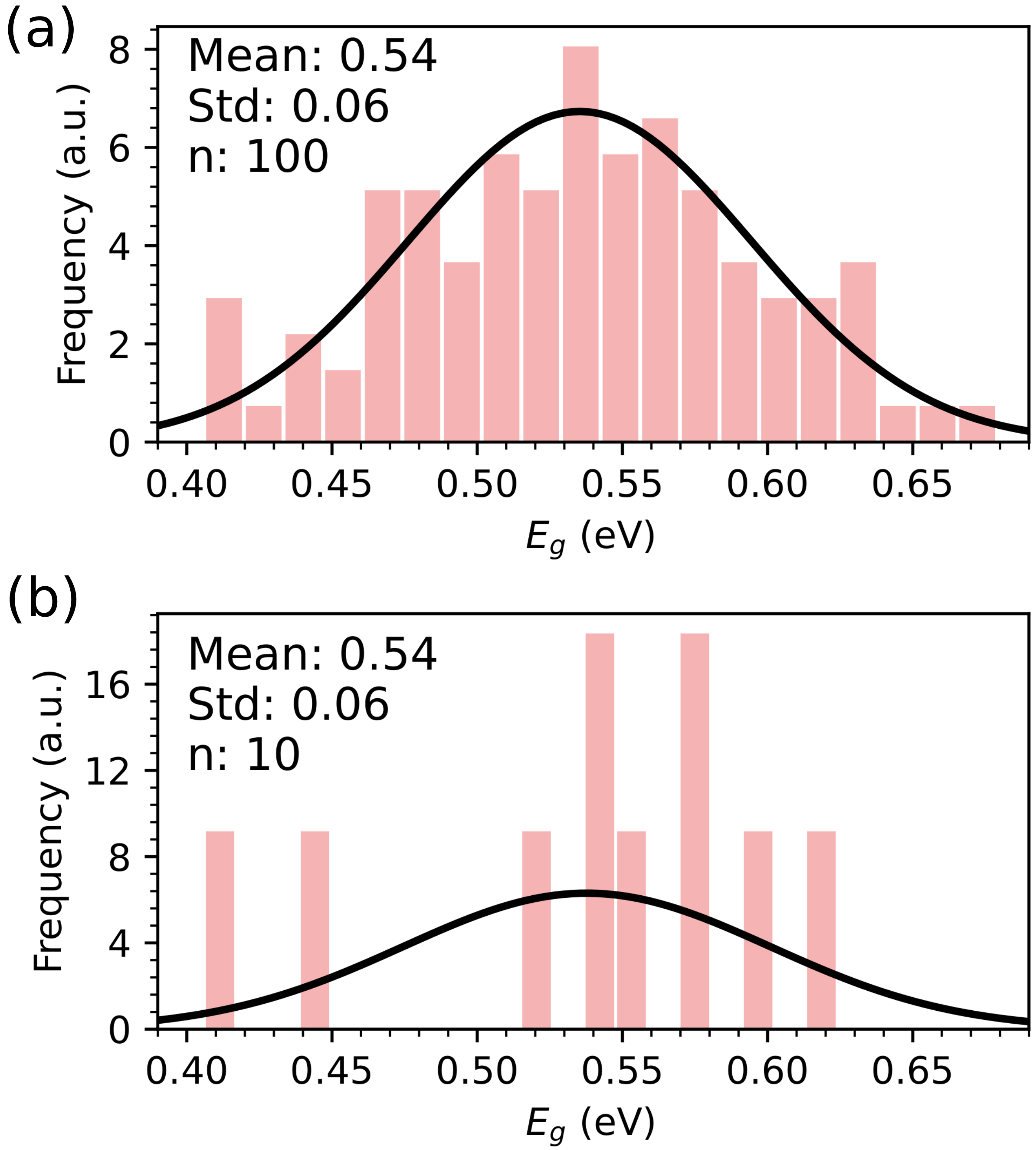

Supplementary Figure 6: Histogram of band gap values computed with PBEsol for (a) $n = 100$ and (b) $n = 10$ states reached with AIMD simulation at $T = 200$ K for the $Pm\bar{3}m$ phase of Ag_3SBr. A fitted Gaussian distribution (black line) along with its mean and standard deviation values are shown in the top left corner of the plot.

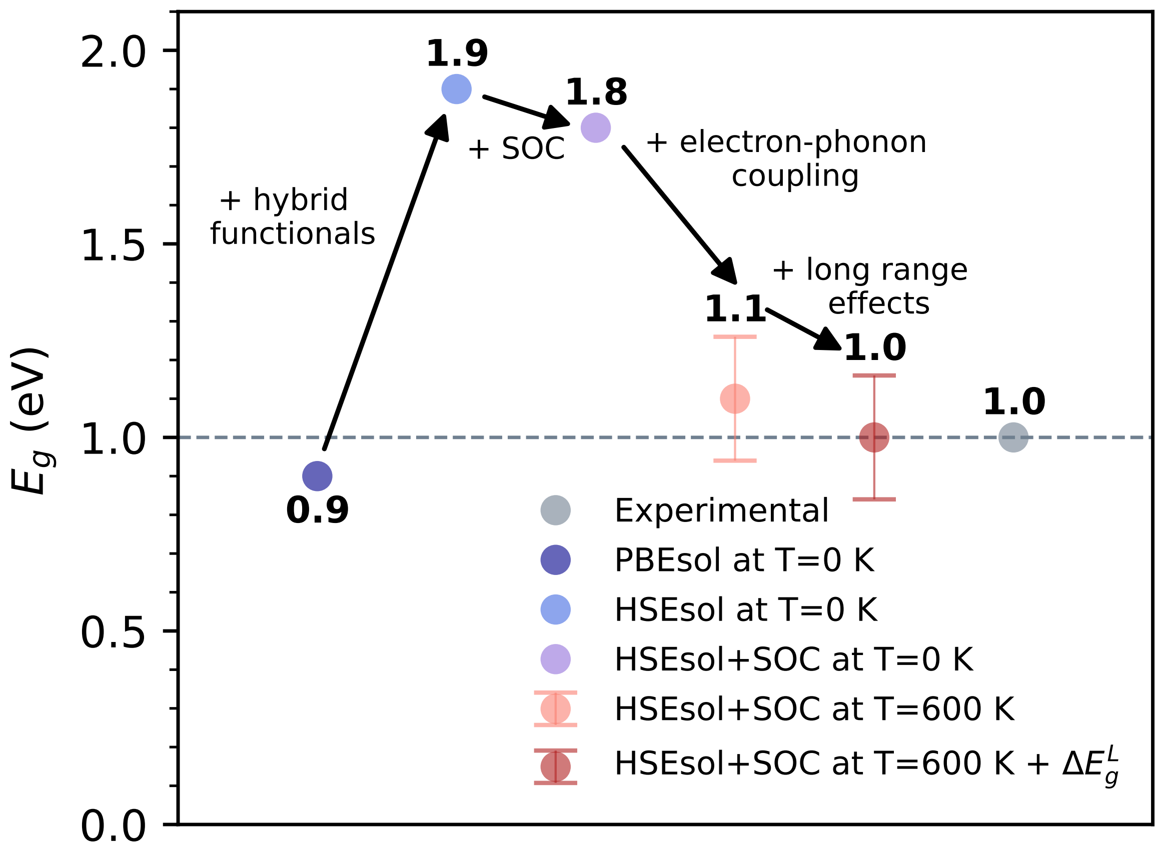

Supplementary Figure 7: Summary of the effect of the employed level of theory on the prediction of the band gap of Ag_3SBr in the cubic $Pm\overline{3}m$ phase, compared with experimental measurements.

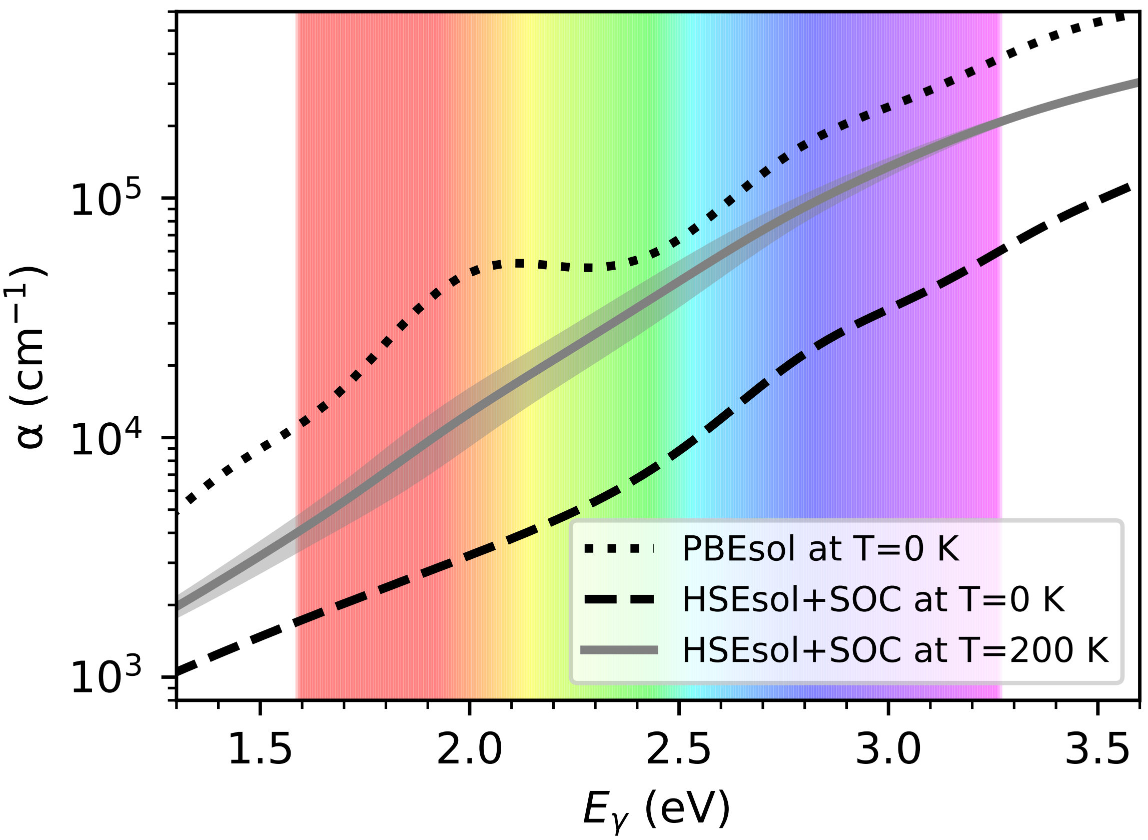

Supplementary Figure 8: Summary of the effect of the employed level of theory on the prediction of the absorption coefficient, α, for Ag_3SBr in the cubic $Pm\overline{3}m$ phase. Results are expressed as function of the photon energy.

V/V_0	ΔE_g (meV)
0.970	90
0.975	87
0.980	60
0.985	45
0.990	31
0.995	15
1.000	0
1.005	-25
1.010	-40
1.015	-50
1.020	-70
1.025	-84
1.030	-86

Supplementary Table I: Band gap variation due to volume expansion and volume contraction in Ag_3SBr.

Functional	AIMD k-point grid	E_g [eV] at 200 K	E_g [eV] at 400 K	E_g [eV] at 600 K
PBEsol	$1 \times 1 \times 1$	0.54 ± 0.06	0.43 ± 0.07	0.39 ± 0.06
PBEsol	$4 \times 4 \times 4$	0.43 ± 0.07	0.40 ± 0.06	0.39 ± 0.05
HSEsol	$1 \times 1 \times 1$	1.37 ± 0.07	1.23 ± 0.08	1.15 ± 0.13
HSEsol	$4 \times 4 \times 4$	1.34 ± 0.09	1.21 ± 0.08	1.10 ± 0.16

Supplementary Table II: Convergence study of the T-induced band gap renormalization. Thermally renormalized band gaps were calculated at $T = 200, 400, 600$ K for Ag_3SBr, using different exchange-correlation functionals and k-points grids in the AIMD simulations.

Functional	Ag_3SBr	Ag_3SI	Ag_3SeBr	Ag_3SeI
PBE	0.85	0.58	0.71	0.49
PBE+SOC	0.77	0.45	0.65	0.36
PBEsol	0.75	0.46	0.61	0.40
PBEsol+SOC	0.68	0.35	0.54	0.28
PBE0	2.61	2.29	2.44	2.15
PBE0+SOC	2.54	2.15	2.39	2.02
HSE06	1.96	1.64	1.80	1.51
HSE06+SOC	1.87	1.49	1.74	1.39
HSEsol	1.86	1.55	1.67	1.40
HSEsol+SOC	1.76	1.40	1.63	1.27
SCAN	1.20	0.91	1.00	0.83
SCAN+SOC	1.14	0.80	0.95	0.71

Supplementary Table III: Theoretical band gaps calculated at $T = 0$ K conditions for CAP using different functionals. E_g values computed at $T = 0$ K for the four studied CAP compounds using different exchange-correlation functionals with and without considering spin-orbit coupling (SOC). Results are expressed in units of eV.

SUPPLEMENTARY METHODS

The crystalline symmetry of phonon-distorted configurations is low, thus performing energy band calculations with a dense enough **k**-points mesh using the HSEsol exchange-correlation functional would be prohibitive in some cases. Thus, we decided to check the difference in electronic bands obtained with the PBEsol and HSEsol exchange-correlation functionals, considering equilibrium and distorted configurations for Ag_3SBr in the cubic $Pm\overline{3}m$ phase. The results of our tests are shown in Supplementary Fig.2. Therein, is observed that the energy difference between bands is underestimated by the PBEsol functional as compared to the HSEsol functional. However, the morphology of the bands, which is of our main interest here, are very similar in both cases. Therefore, for the analysis of frozen-phonon induced band gap variations (Fig.5a in the main text), we decided to use the PBEsol functional.

To perform the thermal renormalization of phonons, we conducted *ab initio* molecular dynamics (AIMD) simulations with $2 \times 2 \times 2$ supercells containing 40 atoms. Considering larger supercells would make the band gap and dielectric tensor computations using HSEsol completely unfeasible. Moreover, the computed band gap with PBEsol for a $4\times4\times4$ supercell with a single **k**-point mesh was determined to be 0.52 ± 0.03 eV, while for a $2\times2\times2$ supercell with the same **k**-point mesh the determined band gap was 0.54 ± 0.06 eV. Thus, we concluded that $2 \times 2 \times 2$ supercells were sufficient for our purposes. To accelerate our computations, we performed the AIMD simulations with a single **k**-point calculation, instead than for a $4 \times 4 \times 4$ **k**-points mesh. This was sufficient to obtain converged renormalized band gaps with temperature, as shown in Supplementary Fig.5 and Supplementary Table II, for both PBEsol and HSEsol exchange-correlation functionals.

The convergence of the short-wavelength phonon band-gap correction term, ΔE_g^S, with the number of configurations, N, was analysed using the PBEsol exchange-correlation functional. Computing the thermal renormalization of the band gap using the HSEsol+SOC approach (SOC stands for spin-orbit coupling) with $N = 10$ was at the limit of our computational capabilities. Supplementary Fig.6 shows that considering $N = 100$ configurations provides exactly the same average band gap value and corresponding standard deviation as considering $N = 10$. Thus, for our present purposes, band-gap averaged values obtained with this number of samples should be sufficient to achieve the required accuracy of 0.1 eV.

Supplementary Fig.7 summarizes the effect of considering different computational approaches on the estimated band gap of Ag_3SBr in the cubic $Pm\overline{3}m$ phase. The need of considering spin-orbit coupling and thermal effects along with the use of hybrid DFT functionals, for achieving consistent agreement with the experimental measurements, is highlighted.

Supplementary Fig.8 shows a similar benchmark study performed for the optical absorption coefficient. The α results obtained with different levels of theory are consistent with the corresponding band gap calculations.

In Supplementary Table III, we present the band gaps computed for the four CAP compounds at $T = 0$ K (under static lattice conditions) using the semilocal GGA functionals PBE and PBEsol, the meta-GGA functional SCAN, and the hybrid functionals HSE06, HSEsol, and PBE0. In all cases, the band gaps are computed with and without considering SOC. The results show that non-hybrid functionals underestimate the band gap while PBE0 overestimates it, as compared to those obtained with the reference hybrid HSEsol functional. Although SOC induces only a small change, it consistently reduces the band gap by approximately 0.1 eV in all the cases.

We also conducted an accuracy test using the GW approximation for the reduced 5-atom cubic CAP unit cell. Compared to the corresponding standard DFT band-gap calculation, we observed a small E_g increase of 0.1 eV, which is within the margin of our reported numerical accuracy. Given that this type of calculation significantly increases computational cost, making it impractical for estimating thermally renormalized band gaps, and does not introduce any substantial changes, we opted not to perform additional GW calculations in this study.
